# The effect of combined transcranial pulsed current stimulation and transcutaneous electrical nerve stimulation on lower limb spasticity in children with spastic cerebral palsy: a randomized and controlled clinical study

**DOI:** 10.1186/s12887-021-02615-1

**Published:** 2021-03-24

**Authors:** Zhenhuan Liu, Shangsheng Dong, Sandra Zhong, Fang Huang, Chuntao Zhang, Yuan Zhou, Haorong Deng

**Affiliations:** 1 Department of Pediatric Rehabilitation, Nanhai Maternity and Children’s Hospital Affiliated to Guangzhou University of Traditional Chinese Medicine, Foshan, Guangdong Province China; 2 Department of Pediatric Rehabilitation, Jiangmen Maternity and Child Health Care Hospital, Jiangmen, Guangdong Province China; 3 Guangzhou Yirui Charitable Foundation, Guangzhou, Guangdong Province China; 4 Department of Pediatric Rehabilitation, Guangzhou City Social Welfare Institute Rehabilitation Hospital, Guangzhou, Guangdong Province China

**Keywords:** Transcranial pulsed current stimulation, TENS, Spastic cerebral palsy, GMFCS levels III–V

## Abstract

**Background:**

In the current study, we applied a combination of non-invasive neuromodulation modalities concurrently with multiple stimulating electrodes. Specifically, we used transcranial pulsed current stimulation (tPCS) and transcutaneous electrical nerve stimulation (TENS) as a novel strategy for improving lower limb spasticity in children with spastic cerebral palsy (SCP) categorized on levels III–V of the Gross Motor Function Classification System (GMFCS) with minimal side effects.

**Methods:**

Sixty-three SCP children aged 2–12 years, who were classified on levels III–V of the GMFCS were randomly assigned to one of two groups, resulting in 32 children in the experimental group and 31 children in the control group. The experimental group underwent a combination therapy of tPCS (400 Hz, 1 mA cerebello-cerebral stimulation) and TENS (400 Hz, max 10 mA) for 30 min, followed by 30 min of physiotherapy five times per week for 12 weeks. The control group underwent physiotherapy only 30 mins per day five times per week for 12 weeks. In total, all groups underwent 60 treatment sessions. The primary outcome measures were the Modified Ashworth Scale (MAS) and Modified Tardieu Scale (MTS). Evaluations were performed 3 days before and after treatment.

**Results:**

We found a significant improvement in MAS and MTS scores of the lower limbs in the experimental group compared to the control group in the hip adductors (Left: *p =* 0.002; Right: *p =* 0.002), hamstrings (Left: *p =* 0.001; Right*: p <* 0.001, and gastrocnemius (Left: *p =* 0.001; Right: *p =* 0.000). Moreover, MTS scores of R1, R2 and R2-R1 in left and right hip adduction, knee joint, and ankle joint all showed significant improvements (*p* ≤ 0.05). Analysis of MAS and MTS scores compared to baseline scores showed significant improvements in the experimental group but declines in the control group.

**Conclusion:**

These results are among the first to demonstrate that a combination of tPCS and TENS can significantly improve lower limb spasticity in SCP children classified on GMFCS levels III–V with minimal side effects, presenting a novel strategy for addressing spasticity challenges in children with severe SCP.

**Trial registration:**

ChiCTR.org, ChiCTR1800020283, Registration: 22 December 2018 (URL: http://www.chictr.org.cn/showproj.aspx?proj=33953).

**Supplementary Information:**

The online version contains supplementary material available at 10.1186/s12887-021-02615-1.

## Background

Spastic cerebral palsy (SCP) is the most common cerebral palsy (CP) subtype, accounting for 77% of all cases of cerebral palsy [1]. SCP, typically presents with increased muscle tone, hyperreflexia, exaggerated deep tendon reflexes, and, in some cases, clonus [2]. Children with SCP who have severe spastic diplegia and spastic quadriplegia are categorized on level III and levels IV–V of the Gross Motor Function Classification System (GMFCS) [3] respectively, and the majority experience significant effects in both legs. Spasticity often results in the development of muscle and joint contractures, torsional deformities of bone, and joint instability at the hip, knee, and ankle [4], which can impact wheelchair positioning, transfers, dressing, and hygiene. Thus, treating lower limb spasticity is an important rehabilitation goal for children with SCP in GMFCS Levels III–V. Current interventions for spasticity include oral medications (e.g., baclofen, tizanidine, and dantrolene [5–7]), physical and occupational therapy (e.g., passive stretching, constraint-induced movement therapy, Bobath therapy, neurodevelopment therapy, massage [8–11]), splinting and casting (i.e., dynamic and static splints that maintain positioning of joints and plaster cast to stretch muscles [12]), botulinum toxin injections, and surgical methods such as selective dorsal rhizotomy and intrathecal baclofen. However, many of the above methods are associated with undesirable side effects and even serious adverse events [13–15]. There is thus a pressing need for the development of new spasticity treatments for children with SCP, with priority given to conservative measures with the fewest side effects.

Non-invasive brain stimulation (NIBS) has been proposed as a possible mechanism to manage spasticity as it can be used repeatedly to target cortical regions, activating or inhibiting neural activity in the cortex [16], which may influence the descending inhibitory input to the dorsal reticulospinal and corticospinal tracts, leading to a reduction of excitatory inputs from the medial reticulospinal and vestibulospinal tracts that ultimately causes spasticity [17, 18]. Upon review of the relevant literature, NIBS studies on CP children that used transcranial direct current stimulation (tDCS) have shown significant improvements in upper limb spasticity, gait, and balance [19–22]. However, these samples did not include severe SCP children of GMFCS levels III–V. To our knowledge, there is only one NIBS study involving children with spastic quadriplegia [23] in which high frequency (5 Hz) repetitive transcranial magnetic stimulation (rTMS) was used to treat upper limb spasticity; however, measured outcomes using Ashworth scale tests failed to reach significance. Moreover, there are not yet studies that have investigated the effects of NIBS on lower limb spasticity in children with SCP in GMFCS levels III–V. Transcranial pulsed current stimulation (tPCS) is a novel type of NIBS that has recently gained increasing attention in experimental settings [24–34], delivering pulsed currents at a predetermined frequency to the cortex, as opposed to the direct current provided by tDCS. While there are yet no studies done involving tPCS and children with SCP, the safety of tPCS had been investigated in the treatment of Parkinson’s disease, with no adverse events recorded, and post-treatment results showed significant improvement in gait and balance [35]. A recent fMRI study [36] observed aberrant functional connectivity within the cerebellum, sensorimotor, left frontoparietal, and salience network in children with SCP when compared to healthy controls. As tPCS in the literature had been demonstrated to reliably induce an enhancement of corticospinal excitability [24, 25], increase the power and connectivity of endogenously generated brain oscillation in a frequency-specific manner [26, 31, 33, 34], and has a facilitatory effect on interhemispheric connectivity [29], this NIBS modality may present a suitable method to address possible pathophysiological mechanism related to SCP.

Transcutaneous electrical nerve stimulation (TENS) is a form of non-invasive peripheral stimulation that has been commonly used in the rehabilitation of children with CP. TENS involves the application of electric currents onto the skin using surface electrodes to target spastic muscles and/or their antagonists [37, 38]. The reduction of spasticity caused by TENS is purportedly due to the massive recruitment of sensory afferents that can suppress motoneuronal excitability through the depression of propriospinal interneurons or the induction of long-term synaptic changes in primary afferents in the dorsal horn [39]. TENS can be applied to the spine and is also known as transcutaneous electrical stimulation of the spine (tsESS) [40–43]. Application of tsESS to the cervicothoracic and thoracolumbar regions has been observed to influence the spinal pathways leading to normalization of spinal reflex hyperexcitability and treatment of hypertonia in subjects with lesions to upper motor neurons [42]. More commonly, TENS is used directly on CP-affected muscles. Several TENS studies have reported positive effects on lower limbs of children with CP that included significant decreases in hip adductor spasticity [44], decreased knee jerk and knee torque impulses [45], and increased walking speed and cadence [46]. However, limitations included small sample sizes (five participants) [45, 46], lack of significant difference in the level of improvement between a one-time trial and with increased sessions of a one-week trial [44], and an inability to attain significant differences in post-treatment Modified Ashworth Scale (MAS) changes and measurement of H-reflex using EMG parameters of the lower limb muscles [46].

A combination of non-invasive neuromodulation (NINM) interventions has been investigated in other medical conditions, such as chronic pain, chronic stroke, and spinal cord injury [47–52], and summative effects had been observed in the induction of corticospinal excitability when transcranial and peripheral stimulation were performed concurrently. These studies reported enhanced treatment benefits that seemed to surpass levels reached by single intervention alone, including improved finger function [47], increased gait ability [48], reduced chronic pain [49, 51, 52], and increased ankle movements [50]. To date, no studies have used a combination of NINMs for the treatment of spasticity in children with CP. However, some studies using Chinese traditional therapies have reported that “Tong Du Xing Shen” acupuncture involving a concurrent stimulation of acupoints on the scalp, governing vessel (spine), and targeted muscles in the lower limbs could significantly improve spasticity and motor function in children with severe SCP [53–56].

### Hypothesis

We hypothesized that a combination of tPCS and TENS, applied concurrently with multiple stimulating electrodes covering the scalp, spine and lower limbs, would be effective in improving lower limb spasticity in children with SCP categorized on GMFCS levels III–V, presenting a novel rehabilitation method with minimal side effects that is safe for the long-term management of spasticity in this population of children.

## Materials and methods

### Ethics statement

A randomized controlled clinical trial was conducted. This study received approval from the Clinical Research Ethics Committee of Guangzhou City Social Welfare Institute Rehabilitation Hospital (process number 20181210) and was carried out in compliance with the ethical standards established in accordance with the Declaration of Helsinki (2013 edition). This study is registered with the Chinese Clinical Trial Registry under registration number ChiCTR1800020283. (URL: http://www.chictr.org.cn/showproj.aspx?proj=33953). Written informed consent was obtained from legal guardians of each participating child.

### Experimental design

**Fig. 1:**
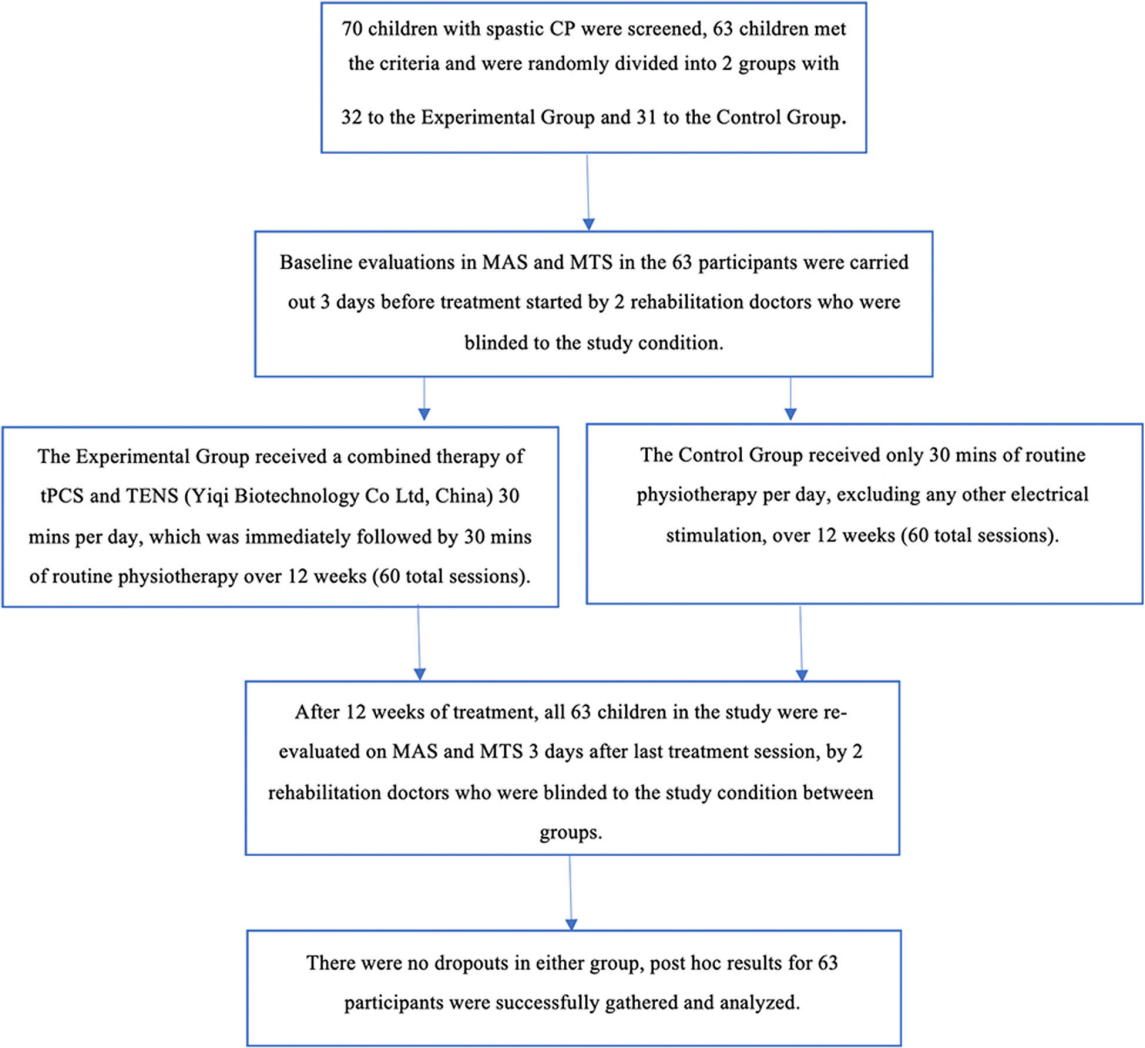
Flowchart of study based on Consolidated Standards of Reporting Trials

The study took place at the Guangzhou City Social Welfare Institute Rehabilitation Hospital, Guangdong province, China, from June 2018 to May 2019. Seventy children with spastic CP were recruited from the Guangzhou City Social Welfare Institute Rehabilitation Hospital and Dongguan City Social Welfare Institute Rehabilitation Center for the trial. Figure 1 presents the CONSORT flow chart of the study. Additional file 1 presents the CONSORT checklist. Additional file 2 presents the trial protocol.

### Inclusion criteria

SCP was diagnosed according to diagnostic criteria of CP found in international guidelines [57]. Inclusion criteria were children with SCP, aged 2–12 years, with GMFCS classification levels of III, IV, or V [3]; lower limb muscle tone in Grades I–IV in accordance to the Modified Ashworth Scale (MAS) [58]; intelligence quotient score > 35 (no worse than moderate intellectual disability) as assessed via Wechsler test scales; no severe psychosocial or behavioral problems, such high aggression or risk of self-harm; no severe cardiopulmonary diseases; and voluntary participation and informed consent signed.

### Exclusion criteria

Initiation of oral antispastic medication, botulinum toxin injections, or surgery performed less than 90 days before enrollment; severe visual or auditory impairment; uncontrollable epilepsy, defined as the occurrence of seizures despite the use of at least one antiepileptic drug at an adequate dose; history of craniotomy or skull defects; severe neurological disorders, such as brain tumors, intracranial infection/ lesions, metal implants in the skull; Severe orthopedic deformities; and immune diseases and skin infections.

### Drop-out criteria

Subjects who voluntarily terminated treatment during the course of treatment; subjects who did not receive treatment according to plan, either due to poor compliance or non-cooperation; and subjects who were not suitable to continue the trial due to serious adverse reactions or appearance of other accompanying diseases.

### Sample size

The number of subjects needed in each study group to test the primary hypothesis was determined based on previous clinical trials assessing spasticity in children with CP. A prior study by Auvichayapat et al. [22] testing the effects of anodal tDCS on upper limb spasticity using the MAS found that a sample size of 46 individuals, divided into two groups (1 mA a-tDCS [*n* = 23], Sham a-tDCS [*n* = 23]), demonstrated an effect with a power of 0.90 and an alpha level of 0.05^.^ If the combined tPCS and TENS therapy in this study had a similar effect on the primary outcome measure of the MAS, the authors determined that 60 participants (30 per condition) would have been sufficient to provide a power of 0.90 with an alpha of 0.05, to which we will add 10 participants to compensate for possible dropouts, totaling 70 participants.

### Randomization and allocation procedures

A simple random sampling method was used to carry out the allocation in accordance with China clinical research standards in “Methodology of Clinical Scientific Research of Integrated Traditional Chinese and Western Medicine (2nd edition).” A number sequence from 1 to 63 was re-ordered by Stata 11.0 software: the first 32 numbers were allocated to the experimental group, and the next 31 numbers were allocated to the control group. Sixty-three numbered placement cards were individually sealed in opaque envelopes and handed over to an administrator uninvolved in the random sampling process, who gave out the envelopes to the 63 subjects entering the trial. The number on the placement card received by each subject would determine their respective allocated group.

### Intervention

**Fig. 2:**
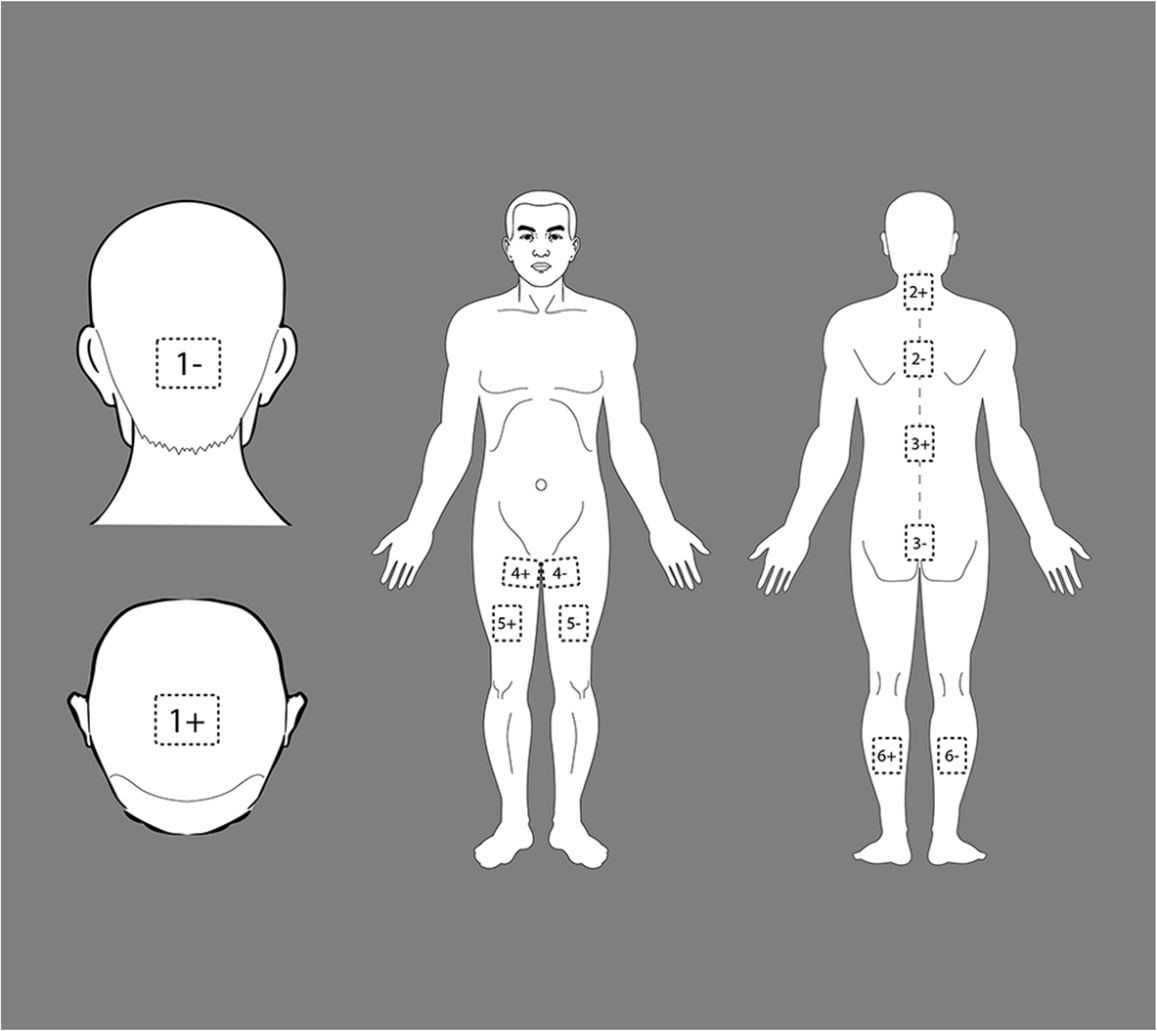
Position of surface gel electrodes during combined tPCS and TENS stimulation. *TENS* transcutaneous electrical nerve stimulation; *tPCS* transcranial pulsed current stimulation

The experimental group (*n* = 32) underwent a combined therapy of tPCS and TENS using a multichannel pulsed current stimulator (YQ-D507; Yiqi Biotechnology Co. Ltd., China) once a day for 30 min, which was immediately followed by 30 min of routine physical therapy. This rehabilitation protocol was performed five times a week (Monday to Friday), for 12 consecutive weeks, totaling 60 sessions. The control group (*n* = 31) was treated with routine physical therapy only, once a day for 30 min, five times a week, for 12 consecutive weeks, totaling 60 sessions. Routine physical therapy primarily consisted of 15 min of passive stretching exercises [59] and 15 min of Chinese “Tui Na” massages [60], which are often used in children with SCP to reduce muscle stiffness, with a focus on lower limb muscles. “Tui Na” massage involved applying oscillating and pressure techniques on meridians and acupoints in the lower limbs to stimulate blockages and knots in the muscles and tendons, thus rebalancing the “Qi” in the body. Passive stretching exercises involved performing manual stretch of the hip, knee, and ankle joints with the child in supine position. The therapist would slowly reach to the end range of motion during flexion and extension of each joint, holding for 40–60 s, and would repeat it five times. The lower extremity muscles that were stretched included the hip flexors, hip extensors, hip adductors, knee flexors, knee extensors, and ankle plantar flexors. Combined tPCS and TENS stimulation was carried out using six pairs of (6 × 9 cm) surface silicone gel electrodes. tPCS involved cerebello-cerebral stimulation, where the anode electrode was positioned over Cz (according to the 10–20 International Electroencephalogram System [61]), covering the Baihui acupoint, and cathode positioned horizontally to cover the cerebellum region, coinciding with Oz (according to the 10–20 International Electroencephalogram System [61]; the bottom edge centered over the inion (Fig. 2, 1+ and 1−). The skin of the scalp was required to be cleaned with saline prior to electrode placement. Stimulating current intensity was set to 1 mA. The second to sixth pairs of electrodes were used for TENS stimulation. The second pair of electrodes was placed on the cervicothoracic region of the spine, with the anode covering C6–C7 and cathode covering T1–T2 [62] (Fig. 2, 2+ and 2−). The third pair of electrodes was placed on the thoracolumbar region, with the anode covering T11–12 and cathode covering L4–L5 [40, 41, 63] (Fig. 2, 3+ and 3−). The fourth pair of electrodes were placed on the adductor longus muscles of the lower limbs (Fig. 2, 4+ and 4−). The fifth pair of electrode pads (Fig. 2, 5+ and 5−) were placed on the rectus femoris muscles of the lower limbs. The sixth pair of electrode pads (Fig. 2, 6+ and 6−) were placed on the gastrocnemius muscles of the lower limbs. The specific strength of the second to sixth pairs of electrodes was adjusted according to the degree of tolerance of individual children, with current intensity varying from 0 to 10 mA.

The output parameters of TPCS were as follows:

- tPCS (first electrode pair) intensity: 1 mA
- TENS (second to sixth electrode pairs) intensity: 0–10 mA
- Pulse width of current (all electrodes): 140 μs
- Frequency (all electrodes): 400 Hz
- Waveform (all electrodes): monophasic unidirectional square pulse

### Assessment

Evaluations were conducted on 63 subjects by two qualified rehabilitation doctors who have been involved in clinical pediatric rehabilitation for more than 5 years. The evaluators were blinded to the study condition of the two groups and did not participate in the treatment of the subjects. Evaluations consisted of Modified Ashworth Scale (MAS) and Modified Tardieu Scale (MTS) as primary outcome measures. Pre-treatment evaluation was done 3 days before first treatment and post-treatment evaluation was done 3 days after the last session of treatment. Both pre- and post-evaluations were conducted in a designated evaluation room that was spacious, quiet, and bright. Each scale was repeatedly measured three times during each evaluation. Each score was the mean of the three measurements.

### *MAS evaluation* [58]

The MAS was used to evaluate muscle spasticity in the lower limbs. MAS evaluation involves the rater manually moving a limb through the range of motion to passively stretch specific muscle groups and a six-point ordinal scale for grading the resistance encountered during such passive muscle stretching. MAS grades of spasticity are as follows: 0 = *normal muscle tone*; 1 = *slight increase in muscle tone, manifested by catch and release or by minimal resistance at the end*; 1+ = *slight increase in muscle tone, manifested by a catch, followed by minimal resistance throughout*; 2 = *more marked increase in muscle tone, but limb easily flexed*; 3 = *considerable increase in muscle tone, passive movement difficult*; and 4 = *limb rigid in flexion or extension*.

### *MTS evaluation* [64, 65]

MTS was used to evaluate the degree of spasticity in the lower limbs of participants. The MTS uses standardized procedures to measure quality of muscle reaction at specified velocities (i.e., fast stretch and slow controlled motion). During the fast stretch, the particular angle at which “catch” [66] occurs from hyperactive stretch reflex is called R1, also known as angle of muscle reaction. During the slow controlled motion, the passive range of motion (PROM) is assessed (called R2), representing the muscle length at rest and recorded as an angle. The difference between the two measures (i.e., R2 − R1; dynamic component of spasticity) is recorded as R. A large difference between R1 and R2 suggests a large dynamic component with a greater capacity for change or improvement. A small difference between R1 and R2 suggests a predominantly fixed contracture in the muscle with a poorer capacity for change.

### Safety monitoring

During the course of study, two experienced pediatric nurses were assigned to systematically observe all participants for adverse reactions such as seizure, nausea, behavioral changes, or severe discomfort. At the end of each treatment session, the children and/or their caregivers in the experimental group were consulted about potential side effects. In the event of any adverse reaction, treatment was immediately terminated, and an Adverse Event related to the procedure was recorded and reported accordingly.

### Statistical methods

SPSS v20.0 was used for statistical analyses. Data were entered into Excel tables by double entry and checked to establish a database. MAS and MTS data were all described in terms of means ± standard deviation. Between these, data of the MAS scales did not conform to the conditions of normal distribution and homogeneity of variance; hence a Mann–Whitney test was performed. The MTS scale data did conform to the conditions of normal distribution and homogeneity of variance; thus, an independent sample *t*-test was used. A Chi-square Test was used to determine any statistical differences between groups in relation to gender, age, height, weight, body mass index (BMI), and/or GMFCS grade before treatment. A *p*-value less than or equal to 0.05 was considered statistically significant.

## Results

A total of 70 children were screened. Of these, five children exhibited severe orthopedic deformities in the lower limbs and two children had taken antispastic medications in the last 90 days, resulting in 63 children meeting inclusion/exclusion criteria, and who were randomly allocated into one of the two groups. Of the 63 children eligible for participation, six exhibited severe spastic diplegia (9.52%), while the remaining 57 exhibited spastic quadriplegia (90.4%). In the first week of treatment, seven children in the experimental group could not tolerate the full intensity of electrical stimulation and the therapist had to adjust to between 50% and 75% of the stated intensity to allow habituation. After 7–10 sessions, all 32 children in the experimental group were accustomed to the combined tPCS and TENS therapy at the stated intensity level and followed through for the remainder of the 60 sessions without incident. All children in the study readily accepted the physical therapy consisting of Tui Na massage and passive stretching; however, a small proportion of children (*n* = 6) who had extremely high lower limb muscle tone cried during passive stretching during the first 3 weeks. These children subsequently stopped crying when their lower limb spasticity progressively improved. All 63 children completed the study with no dropouts.

Table 1 displays the demographic characteristics and GMFCS classification of the participants in the two groups. Chi-square test analyses showed no significant difference between the two groups at baseline with respect to GMFCS grade (*p* = 0.57), weight (*p* = 0.09), BMI (*p* = 0.66), sex (*p* = 0.25). An independent sample *t*-test for age (*p* = 0.01) and height (*p* = 0.02) were significant at *p* ≤ 0.05, but the authors felt these were unlikely to bias study outcomes given that a wide age range was included in the study and the difference in average age and height across the experimental and control groups remain insignificant at *p* > 0.01 (see Table 1 for details). Baseline *p*-values of MAS and MTS between the two groups were not statistically significant, suggesting similar disease severity in both groups. (See “Before treatment *p*-values” in Tables 2 and 4).

**Table 1:** Demographic characteristics and Gross Motor Function Classification System (GMFCS) levels of participants

Item	Treatment group (total: 32 participants)	Control group (total: 31 participants)	***t***/χ^**2**^	***p***
Sex (F/M)	9/23	13/18	1.321	0.250
Age (years)	7.63 ± 2.459	9.19 ± 2.315	−2.605	0.012
Height (cm)	100.062 ± 11.725	106.483 ± 9.807	2.354	0.022
Weight (kg)	15.403 ± 4.854	17.755 ± 6.071	1.701	0.094
BMI	15.150 ± 2.384	15.555 ± 4.667	0.437	0.664
GMFCS (III/IV/V)	5/15/12	6/10/15	1.103	0.576

*BMI* Body mass index, *F* Female, *M* Male

**Table 2:** Modified Ashworth Scale (MAS) scores between the two groups before and after treatment

Item	Experimental group (32 cases)		Control group (31 cases)		Before treatment	After treatment
	Baseline	Post-treatment	Baseline	Post-treatment	Z/***p***	Z/***p***
Adductor						
Left	2.29 ± 1.56	1.10 *±* 0.68	1.93 *±* 1.174	2.37 *±* 1.43	0.718/0.473	−4.395/0.002
Right	2.29 *±* 1.485	1.01 *±* 0.58	1.98 *±* 1.18	2.41 *±* 1.44	0.581/0.561	−5.521/0.002
Hamstring tendon						
Left	2.10 *±* 1.29	1.07 *±* 0.58	1.71 *±* 0.87	2.25 *±* 1.04	0.759/0.448	−5.810/0.001
Right	2.06 *±* 1.22	1.06 *±* 0.61	1.82 *±* 0.86	2.32 *±* 1.05	0.352/0.725	−6.022/0.000
Gastrocnemius						
Left	2.84 *±* 1.18	2.00 *±* 0.89	2.59 *±* 1.11	3.19 *±* 1.13	0.817/0.414	−6.564/0.001
Right	2.84 *±* 1.24	2.03 *±* 0.98	2.69 *±* 1.18	3.33 *±* 1.12	0.537/0.592	−6.647/0.000

### Comparative analysis of MAS

**Fig. 3:**
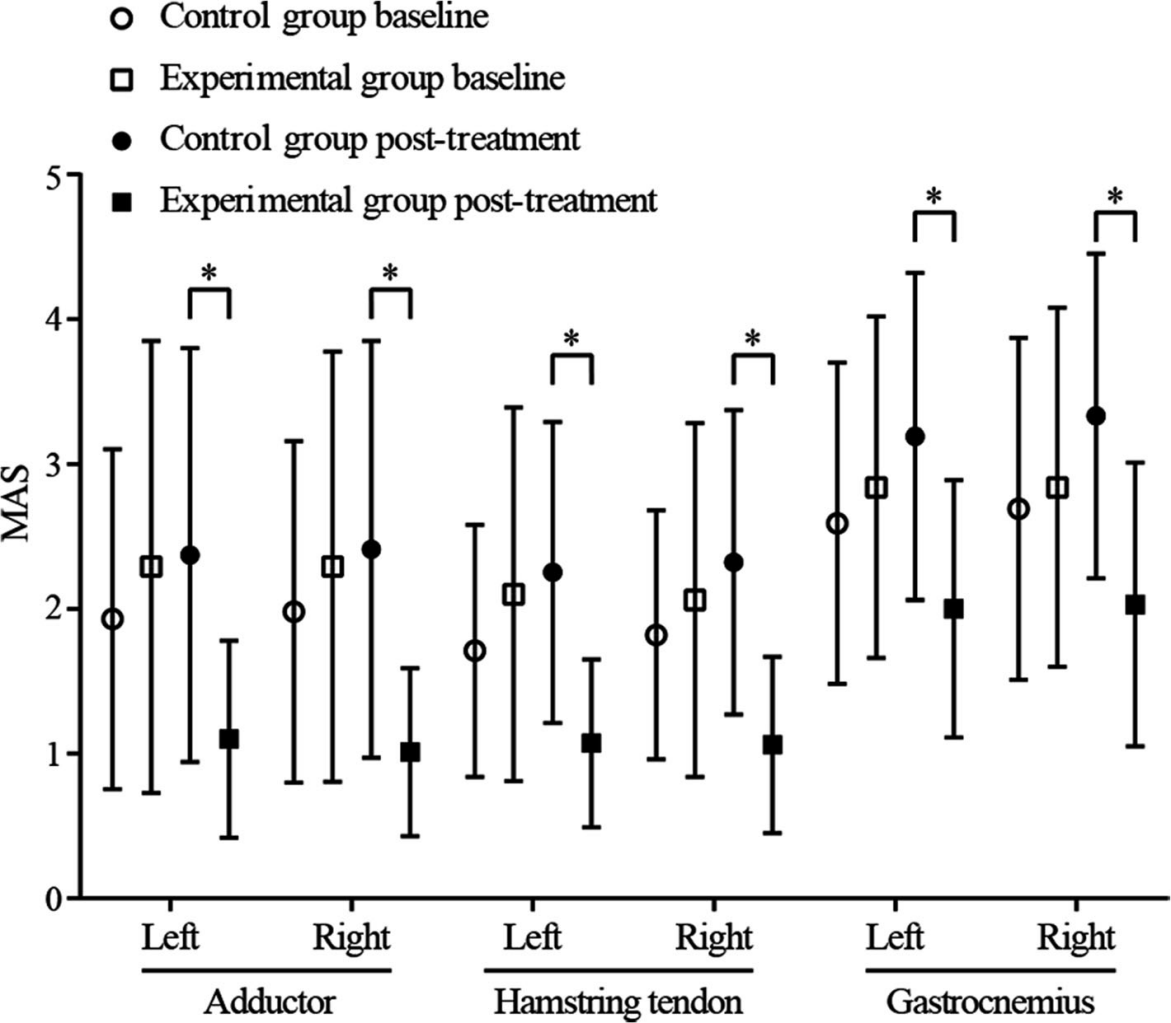
Comparative analysis of MAS in the two groups before and after treatment. *MAS* Modified Ashworth Scale

Post treatment, there was a statistically significant decrease in MAS scores in the hip adductors (L: *p =* 0.002; R: *p =* 0.002), hamstrings (L: *p =* 0.001; R: *p =* 0.000), and gastrocnemius (L: *p =* 0.001; R: *p =* 0.000) in the experimental group when compared to the control group. See Table 2 (“After-treatment *p*-values”) for details and Fig. 3 for a summary of comparative analyses of pre-and post-treatment MAS scores. In the experimental group, within-group analysis of post-treatment MAS scores compared to baseline showed significant improvements for the hip adductors (L: *p =* 0.002; R: *p =* 0.000), hamstrings (L: *p =* 0.000; R: *p =* 0.000), and gastrocnemius (L: *p =* 0.007; R: *p =* 0.008). However, in the control group, we observed a significant decline from baseline to post-treatment MAS scores, with significant declines observed in the gastrocnemius (L: *p =* 0.037; R: *p =* 0.023) and left hamstring tendon (*p* = 0.037). See Table 3 (“Pre vs Post *p*-values”) for details.

**Table 3:** Intra-group Modified Ashworth Scale (MAS) scores pre and post treatment

Item	Experimental group (32 cases)		Control group (31 cases)		Experimental group (pre vs post)	Control group (pre vs post)
	Baseline	Post-treatment	Baseline	Post-treatment	Z/***p***	Z/***p***
Adductor						
Left	2.29 ± 1.56	1.10 ± 0.68	1.93 ± 1.174	2.37 ± 1.43	−3.172/0.002	−1.313/0.189
Right	2.29 ± 1.485	1.01 ± 0.58	1.98 ± 1.18	2.41 ± 1.44	− 3.788/0.000	− 1.335/0.182
Hamstring tendon						
Left	2.10 ± 1.29	1.07 ± 0.58	1.71 ± 0.87	2.25 ± 1.04	− 3.905/0.000	−2.084/0.037
Right	2.06 ± 1.22	1.06 ± 0.61	1.82 ± 0.86	2.32 ± 1.05	− 3.964/0.000	−1.936/0.053
Gastrocnemius						
Left	2.84 ± 1.18	2.00 ± 0.89	2.59 ± 1.11	3.19 ± 1.13	−2.677/0.007	−2.081/0.037
Right	2.84 ± 1.24	2.03 ± 0.98	2.69 ± 1.18	3.33 ± 1.12	− 2.672/0.008	− 2.269/0.023

### Comparative analysis of MTS scores

**Fig. 4:**
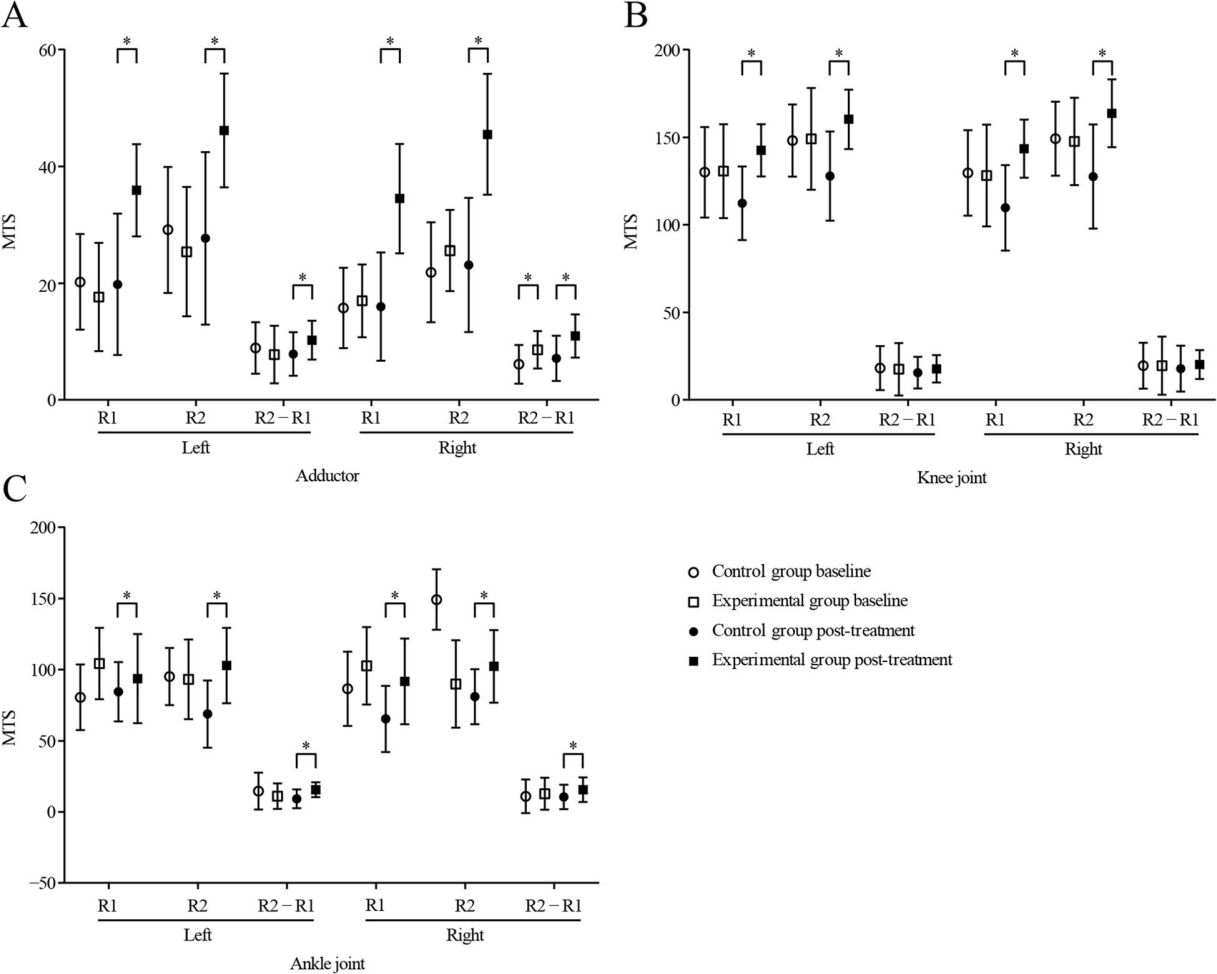
Comparative analysis of MTS scores in the two groups before and after treatment. *MTS* Modified Tardieu Scale

Post treatment, comparisons between the experimental group and the control group showed significant improvements in R1 (fast stretch muscle response) and R2 (passive range of motion) of left and right hip adduction, knee joint, and ankle joint. Post-treatment comparisons of R2-R1 scores of the left and right hip adduction and ankle joints also significantly improved, with the exception of R2-R1 scores of the left and right knee joints, which improved but did not manage to reach significant levels (L: *p =* 0.306; R: *p =* 0.397). See Table 4 (“After-treatment *p*-values”) for details and Fig. 4 for a summary of comparative analysis of pre- and post-treatment MTS scores. MTS scores in the experimental group significantly improved post-treatment compared to baseline, with the exception of the R2-R1 scores of the knee joints (L: *p =* 0.910; R: *p =* 0.827) and right ankle joint (R: *p =* 0.141), which improved from baseline but did not reach significant levels. However, in the control group, we observed significant declines in both knee joints and the left ankle joint from baseline to post-treatment (see Table 5 (“Pre vs Post *p*-values”) for details).

**Table 4:** Modified Tardieu Scale (MTS) scores between experimental and control groups pre- and post- treatment

Item	Experimental group		Control group		Before treatment	After treatment
	Baseline	Post − treatment	Baseline	Post-treatment	***t***/***p***	***t***/***p***
Adductor (left)						
R1	17.63 ± 9.27	35.91 ± 7.90	20.23 ± 8.19	19.81 ± 12.10	−1.178/0.243	6.269/0.000
R2	25.41 ± 11.06	46.16 ± 9.73	29.13 ± 10.78	27.68 ± 14.77	−1.352/0.182	5.880/0.000
R2-R1	7.78 ± 4.93	10.25 ± 3.34	8.90 ± 4.42	7.87 ± 3.74	−0.949/0.346	2.661/0.010
Adductor (right)						
R1	17.00 ± 6.24	34.50 ± 9.36	15.77 ± 6.90	16.00 ± 9.29	0.739/0.462	7.868/0.000
R2	25.59 ± 6.94	45.47 ± 10.34	21.87 ± 8.55	23.13 ± 11.49	1.900/0.062	8.114/0.000
R2-R1	8.59 ± 3.21	10.97 ± 3.70	6.10 ± 3.32	7.13 ± 3.86	3.034/0.004	4.029/0.000
Knee joint (left)						
R1	130.66 ± 26.81	142.56 ± 14.91	130.03 ± 25.86	112.26 ± 21.08	0.094/0.925	6.602/0.000
R2	148.13 ± 21.87	160.31 ± 17.03	148.16 ± 20.61	127.81 ± 25.50	−0.007/0.995	5.966/0.000
R2-R1	17.47 ± 14.93	17.75 ± 7.81	18.13 ± 12.61	15.55 ± 9.09	−0.189/0.851	1.032/0.306
Knee joint (right)						
R1	128.13 ± 29.12	143.53 ± 16.61	129.65 ± 24.42	109.74 ± 24.38	0.224/0.823	6.445/0.000
R2	147.66 ± 24.98	163.69 ± 19.34	149.19 ± 21.18	127.55 ± 29.82	−0.263/0.793	5.724/0.000
R2-R1	19.53 ± 16.62	20.16 ± 8.27	19.55 ± 13.11	17.81 ± 13.13	−0.005/0.996	0.852/0.397
Ankle joint (left)						
R1	104.22 ± 25.18	93.71 ± 31.38	80.48 ± 23.143	84.38 ± 20.93	1.948/0.056	−3.578/0.001
R2	93.13 ± 28.048	102.90 ± 26.54	95.16 ± 20.10	68.75 ± 23.55	1.575/0.121	−3.081/0.003
R2-R1	11.09 ± 8.956	15.63 ± 5.198	14.68 ± 12.970	9.19 ± 6.59	1.280/0.206	4.306/0.000
Ankle joint (right)						
R1	102.66 ± 27.20	91.68 ± 30.164	86.45 ± 26.115	65.31 ± 23.13	0.827/0.411	−3.900/0.00
R2	89.84 ± 30.756	102.26 ± 25.52	149.19 ± 21.18	80.94 ± 19.40	0.471/0.639	−3.740/0.00
R2-R1	12.81 ± 11.284	15.63 ± 8.684	10.97 ± 11.862	10.58 ± 8.590	0.633/0.529	2.317/0.024

**Table 5:** Modified Tardieu Scale (MTS) scores across groups pre- and post- treatment

Item	Experimental group		Control group		Experimental group (Pre vs Post)	Control group (Pre vs Post)
	Baseline	Post-treatment	Baseline	Post-treatment	***t***/***p***	***t***/***p***
Adductor (left)						
R1	17.63 ± 9.27	35.91 ± 7.90	20.23 ± 8.19	19.81 ± 12.10	−8.996/0.000	0.190/0.851
R2	25.41 ± 11.06	46.16 ± 9.73	29.13 ± 10.78	27.68 ± 14.77	−9.777/0.000	0.538/0.594
R2-R1	7.78 ± 4.93	10.25 ± 3.34	8.90 ± 4.42	7.87 ± 3.74	−2.760/0.010	1.057/0.299
Adductor (right)						
R1	17.00 ± 6.24	34.50 ± 9.36	15.77 ± 6.90	16.00 ± 9.29	−8.930/0.000	−0.125/0.901
R2	25.59 ± 6.94	45.47 ± 10.34	21.87 ± 8.55	23.13 ± 11.49	−9.667/0.000	−0.594/0.557
R2-R1	8.59 ± 3.21	10.97 ± 3.70	6.10 ± 3.32	7.13 ± 3.86	−3.263/0.003	−1.132/0.266
Knee joint (left)						
R1	130.66 ± 26.81	142.56 ± 14.91	130.03 ± 25.86	112.26 ± 21.08	−1.893/0.068	3.481/0.002
R2	148.13 ± 21.87	160.31 ± 17.03	148.16 ± 20.61	127.81 ± 25.50	−1.989/0.056	3.743/0.001
R2-R1	17.47 ± 14.93	17.75 ± 7.81	18.13 ± 12.61	15.55 ± 9.09	−0.114/0.910	1.154/0.258
Knee joint (right)						
R1	128.13 ± 29.12	143.53 ± 16.61	129.65 ± 24.42	109.74 ± 24.38	4.486/0.045	4.386/0.000
R2	147.66 ± 24.98	163.69 ± 19.34	149.19 ± 21.18	127.55 ± 29.82	−2.284/0.029	3.996/0.000
R2-R1	19.53 ± 16.62	20.16 ± 8.27	19.55 ± 13.11	17.81 ± 13.13	−0.221/0.827	0.637/0.529
Ankle joint (left)						
R1	93.12 ± 28.05	68.75 ± 23.55	80.48 ± 23.143	93.71 ± 31.38	3.665/0.001	−3.561/0.001
R2	104.22 ± 25.18	84.38 ± 20.94	95.16 ± 20.10	102.90 ± 26.55	4.282/0.000	−2.179/0.037
R2-R1	11.09 ± 8.956	15.63 ± 5.198	14.68 ± 12.970	9.19 ± 6.59	−3.177/0.003	2.761/0.010
Ankle joint (right)						
R1	89.84 ± 30.77	65.31 ± 2314	86.45 ± 26.115	91.68 ± 30.16	3.994/0.000	−1.183/0.246
R2	102.66 ± 27.20	80.94 ± 19.40	97.42 ± 22.76	102.26 ± 25.52	4.486/0.000	−1.311/0.200
R2-R1	12.81 ± 11.284	15.63 ± 8.684	10.97 ± 11.862	10.58 ± 8.590	− 1.509/0.141	0.162/0.873

### Adverse reactions

In the course of the study, only mild skin redness (*n* = 3) at the electrode sites were reported. No participant from either group withdrew from the research due to adverse reactions.

## Discussion

In the present study, all of the participants belonged to GMFCS levels III–V, with 57 of the 63 children (90.4%) exhibiting spastic quadriplegia. The level of spasticity in both extremities observed in our study participants was severe, similar to other reports on children with SCP and spastic quadriplegia [67]. Although most of the current literature emphasizes the treatment of spasticity mainly when it adversely impacts daily functioning [68], this may not be a practical consideration for children with severe SCP. The frequent existence of comorbidities, such as severe intellectual, cognitive, and sensory impairments [69] in this category hinders the processing and learning of new motor skills; therefore, major improvement in functionality is rare. Our results showed that it would be more beneficial to target spasticity reduction solely as a rehabilitation goal for children with severe SCP even if it did not carry over to more functional activity, for purposes of improving comfort, reducing pain, and easing the burdens of their caregivers. However, current available spasticity treatment options invariably have undesirable side effects, and management of spasticity in this sub-category of SCP children is challenging.

NINM is an emerging class of treatment that has been employed to modulate neural circuitry plasticity in the brain and the spinein an attempt to foster neuro-recovery processes with a potential effect on spasticity [70, 71]. One of the key benefits of NINM is having very minimal side effects relative to pharmacological and surgical options [72], making it an attractive treatment option for children with SCP. Here, we attempted to explore a new NINM alternative, specifically via the combination of tPCS on the cortex and TENS on the spine and targeted muscles in the lower limbs, for the treatment of lower limb spasticity in children with SCP categorized on GMFCS levels III–V. Results of our study showed this combination of NINM modalities was highly effective in improving lower limb spasticity in children with SCP categorized as GMFCS levels III, as evaluated by MAS and MTS scores.

Prior literature has shown that when NIBS modalities such as tDCS were used as single intervention in the treatment of spasticity in SCP children, it seemed to have greater effects in proximal than distal muscles [73], with more reports of improvement in spasticity in the upper limbs than in the lower limbs. It is worth noting that related NIBS studies mostly sampled ambulant CP children already functioning at higher levels (categorized on GMFCS levels I–II), and the only rTMS study that had included spastic quadriplegia children [23] failed to attain significant levels in post-treatment MAS scores evaluating upper limb spasticity. Given the modest effect of tDCS on spasticity and limited evidence of effectiveness in rTMS for a more severe SCP population, we were inclined towards choosing tPCS as the transcranial stimulation modality in our study. Support for tPCS included a previous head modeling study [74] that documented tPCS could reach deeper brain regions, such as the midbrain, pons, insula, thalamus, and hypothalamus when compared with tDCS, while the latter seemed to mainly increase cortical excitability under the stimulating electrodes [31]. Additionally, several studies also reported that tPCS could influence interhemispheric and functional connectivity within brain networks [29, 31, 33, 34] and thus may be suitable for addressing aberrant functional connectivity related to possible pathophysiological mechanism observed in SCP [36].

Spasticity in CP is reported to be caused by a loss or reduction of the inhibitory influences conducted by the dorsal reticulospinal tract to circuits in the spinal cord, increasing the excitability of gamma and alpha motoneurons [37, 75]. The dorsal reticulospinal tract originates in the ventromedial bulbar reticular formation, which is a powerful inhibitory area of muscle activity directly influenced by the premotor cortex [76]. By applying tPCS to the cortex, the resulting enhancement of corticospinal excitability may facilitate influences on the ventromedial bulbar reticular formation and the dorsal reticulospinal tract, leading to enhanced effects in spinal inhibitory circuits. However, in the case of children with severe SCP, due to the frequent existence of high disruption in the relay of signals between the pyramidal tract and the peripheral nervous system [77], increased inhibitory influences produced by tPCS may not be optimally transmitted down the descending spinal pathways, as corticospinal neurons may not be able to synapse efficiently onto alpha motor neurons. Therefore, we added TENS at the spine and lower limbs for the stimulation of cutaneous afferents that has been reported to directly suppress alpha motoneuronal excitability through depressing the propriospinal interneurons or inducing long-term synaptic changes in primary afferents in the dorsal horn [39, 78].

Post-treatment, the experimental group showed marked improvement in passive stretch resistance in the hip abductors, hamstrings, and gastrocnemius as measured by decreased MAS scores, and significant improvement in passive range of motion of the hip adductor, knee joint, and ankle joint as measured by MTS. Positive changes in lower limb spasticity in the experimental group were significant when compared to the control group and also significant when compared to baseline scores within the same group. Our results were in stark contrast with existing NIBS-alone and TENS-alone studies related to the treatment of spasticity in children with SCP, where improvements could not reach significant levels as measured by post-treatment MAS scores [23, 46] or measurement of H-reflex using EMG parameters [46], despite having study samples which exhibited less severe SCP than in our study. Our positive post-treatment study outcomes were consistent with the findings in other studies that combined transcranial and peripheral stimulation using NINMs for the treatment of chronic pain, chronic stroke, and spinal cord injury [47–52], where enhanced treatment outcomes have been reported that surpassed levels reached by single intervention alone.

We also observed a steady accumulation of positive changes in lower limb spasticity in the experimental group during the 12-week study, in contrast to a previous study that reported that the level of improvement in spasticity showed no significant difference between a one-session stimulation and a one-week stimulation [44]. It was possible that the accumulation-up of improvements in lower limb spasticity over the course of our study was due to the induction of prolonged neuroplastic and spinal plasticity changes, which were precipitated by an enhancement of corticospinal excitability following the combined tPCS and TENS therapy; this was consistent with findings in other studies that combined transcranial and peripheral stimulation using NINMs [47, 49–51].

The control group showed deterioration across board from baseline to post-treatment. However, given that baseline evaluations between groups were not significant, it is reasonable to conclude that lower limb spasticity improvements in the experimental group were attributable to the addition of the combined tPCS and TENS therapy, rather than a particular decline in control group scores. These results could have been due to children in our study having very severe muscle contractures and musculoskeletal deformities even at baseline, and that physical therapy methods consisting of passive stretching and massage were insufficient to slow down the progress of deterioration in clinical conditions at this level of severity. Indeed, this assumption was supported by findings of a study by Fragala et al. [79], who reported a lack of consistent patterns of gains or loss in the passive range of motion of the lower extremity after physical therapy intervention in spastic quadriplegia children categorized on GMFCS Levels IV and V. In addition, Hanna et al. [80] reported in their longitudinal study that children with CP categorized on GMFCS levels III–V typically experienced a significant decline in motor function after the average ages of 6–7 years, which was not observed in children with CP of GMFCS Levels I and II. Thus, the declines may have been due to a naturally occurring deterioration commonly associated with this sub-category of severe SCP.

We utilized multiple pairs of stimulating electrodes, similar to a previous study that used Acupuncture needles [53] for the treatment of children with CP. Children with spastic quadriplegia (GMFCS IV–V) suffered damage to both sides of the brain, and the relay of signals between the pyramidal tract and the peripheral nervous system is highly disrupted due to inappropriately organized neuromuscular junctions developed as an adaptive response to the years of altered activity, weakness, poor coordination, and spasticity [77]. Given that they made up the majority of our study sample, the rationale of applying multiple pairs of electrodes to stimulate the scalp, spine, and lower limbs concurrently, hence, was an attempt to facilitate neurotransmission to modulate spinal inhibitory circuits and induce corticospinal excitability changes [81–83]. Specifically, tPCS cathode electrode was positioned to cover the cerebellum region of the cortex, following evidence that cathodal cerebellar stimulation could lead to the correction of cerebellar overactivity and produce inhibitory effects that could improve lower limb spasticity [84]. On the spine, we applied two pairs of electrodes on the cervicothoracic and thoracolumbar regions following evidence in transpinal studies [42, 43] that stimulating these regions could affect ipsilateral and contralateral actions of corticospinal neurons to enhance corticospinal excitability; On the lower limbs, three pairs of 400 Hz TENS electrodes were applied directly on agonist spastic muscles (adductor longus, rectus femoris, and gastrocnemius), supported by evidence that high frequency (≥ 99 Hz) TENS on the periphery could recruit larger diameter afferent during stimulation to relieve spasticity that was accompanied by a decrease on H-reflex amplitude, which was not observed when lower frequencies (< 50 Hz) were used [75, 85, 86]. In fact, 400 Hz stimulation frequency was applied for all six pairs of electrodes to maximize force enhancement during stimulation to increase effects on corticospinal neuromodulation, following evidence [87] that observed the induced force enhancement during tPCS stimulation was most highly correlated with higher order power harmonics of the stimulation waveform at 400–480 Hz.

While it is difficult to speculate how exactly tPCS and TENS may have interacted to produce a superior clinical benefit in reducing lower limb spasticity in children with severe SCP, the rationale for combining these two therapies was. Whereas tPCS would exert its effect through the modulation of cortical structures that led to an increase in descending inhibitory signals, TENS on the spine and lower limbs would modulate afferent signaling in the peripheral nerves and spinal cord, which would further suppress the portion of motoneuronal excitability that had been bypassed by unsuccessful relay of efferent inhibitory signals due to disruptions in the descending spinal pathways.

In conclusion, post-treatment differences in MAS and MTS scores between the experimental and control groups indicated that the combination therapy of tPCS and TENS, applied with a multiple electrode methodology, is an effective treatment mode for lower limb spasticity in children with SCP categorized on GMFCS levels III–V. As previous studies had only produced modest treatment effects on the lower limbs of less severe SCP children, positive results in our study suggest that a combination of transcranial and peripheral stimulation is likely to be more efficacious in treating spasticity, compared to a single intervention of NIBS or TENS. Finally, minimal side effects associated with tPCS and TENS would be highly beneficial to SCP children by providing a safe alternative to manage long-term spasticity, helping to improve comfort, delay the progression of musculoskeletal deformities, and ease the burdens of their caregivers.

### Safety considerations

In the present study, safety issues were identified a priori by the authors who, on average, have over 20 years of pediatric clinical experience in China. The tPCS used for cortical stimulation in the study belonged to the category of low-intensity transcranial electrical stimulation and no serious adverse events (SAEs) have been reported so far in over 18,000 sessions administered to healthy subjects, as well as in neurological and psychiatric patients. Moderate adverse events (AEs), as defined by the necessity to intervene, are rare, and include skin burns due to suboptimal electrode-skin contact. Mild side effects of transcranial electrical stimulation (tES) such as itching, tingling, burning sensations, and transient redness may occur during treatment [88–92].

For safety of tES on children, the recommended dose needs to compensate for thinner skull and lower resistance [93, 94]. Mattai et al. [95] explored the safety and tolerability of tDCS in children with childhood-onset schizophrenia and found that 10 sessions of 2 mA tDCS for 20 min, 25 cm electrodes, were administered without incident in the test subjects with no serious side effects. Furthermore, a study by Jaberzadeh that compared tPCS to tDCS showed that participants tolerated a-tPCS better than the conventional a-tDCS [24]. In the present study, tPCS (unidirectional monophasic pulse square wave) was controlled at 1 mA, 30 min per session, which is within the confines of conventional tES and safe for children. In addition, our study design passed the safety review conducted by Guangzhou City Social Welfare Institute Rehabilitation Hospital Ethics committee, who gave approval for this study.

### Limitations of study

This study had important limitations that need to be discussed. First, we did not conduct any comparison with sham stimulation, so the placebo effect cannot be excluded. However, we believe that it was unlikely, because in the current study, the severe cognitive deficits of the participants made them blind to treatment condition for all intents and purposes. It was noted that participants in our study were mostly spastic quadriplegia categorized as levels GMFCS IV and V and were between 7 and 9 years old. Further, the severity of their clinical conditions had been present for many years and it would be rather inconceivable that placebo effects alone would have mediated the improvements in lower limb spasticity observed in the experimental group in a period of 12 weeks. The two doctors who were responsible for evaluating the impact of the procedures were also blinded to which participant belonged to the Experimental or control groups. Second, there was no active TENS-alone group (sham tPCS/active TENS) or tPCS-alone group (active tPCS/sham TENS). Therefore, we cannot rule out that the superior effects of the combination therapy of tPCS and TENS were just because of either tPCS or TENS alone. Although this alternative explanation needed to be considered, it was less likely, given that other NIBS-alone [19–23] and TENS-alone [44–46] studies have reported less positive results compared to our study in children with SCP who were less severe than our sample children. Third, due to limited resources, we did not evaluate changes using clinical diagnostic instruments such as EMG, TMS, FMRI or high-density EEG. Thus, we are unable to identify the exact contribution of each electrode or the interaction effects among them to the final positive outcome, and further studies would be needed to investigate this aspect. Finally, post-treatment evaluations were performed 3 days after the last session of treatment and not at any other timepoint. Therefore, we were not able to evaluate the longer-term effects of the combination therapy of tPCS and TENS.

## Conclusions

To the best of our knowledge, the present study was the first to evaluate the effects of a combination of NINM approaches, specifically tPCS and TENS, for the treatment of lower limb spasticity in children with SCP categorized on GMFCS levels III–V. Post-treatment inter-group comparison of MAS and MTS scores showed statistically significant differences, indicating that the combination therapy of tPCS and TENS, applied with a multiple electrode methodology covering the scalp, spine, and lower limb, was effective for improving lower limb spasticity in children with SCP categorized on GMFCS levels III–V. As previous NIBS-alone and TENS-alone studies had produced modest treatment effects on the lower limbs of less severe SCP children, positive results in the present study would further suggest that a combination of transcranial and peripheral stimulation was more efficacious than a single intervention of NIBS or TENS in the treatment of lower limb spasticity, especially in a more severe SCP population. Our relatively large study sample size of 63 children also gave strong validation to our results compared to smaller samples of 5–10 children in other related studies in the literature. Minimal side effects associated with tPCS and TENS would present this combination therapy as a novel and safe alternative for SCP children to manage long-term spasticity, ensuring greater comfort, pain reduction, a delay in the progression of musculoskeletal deformities, and easing the burdens of their caregivers. Further studies would be needed to confirm these results by using clinical diagnostic instruments, such as high-density EEG, EMG, TMS, or fMRI. Future investigations that include the possibility of performing post-treatment evaluations at other timepoints, such as before or after 12 weeks, and with the addition of tPCS-alone and TENS-alone control groups, would contribute to a more in-depth evaluation of the effects of the combination therapy of tPCS and TENS.

## Supplementary Information

**Additional file 1.** CONSORT Checklist.

**Additional file 2.** Trial Protocol.

## Data Availability

The datasets supporting the conclusions of this article are included within the article and its additional files. The original data of individual participants generated and analyzed during the current study are available in the ResMan repository of Clinical Trial Management Public Platform: http://www.medresman.org.cn/uc/projectsh/projectedit.aspx?proj=1048.

## Abbreviations

AEs: Adverse events
BMI: Body mass index
F: Female
GMFCS: Gross Motor Function Classification System
M: Male
MAS: Modified Ashworth Scale
MTS: Modified Tardieu Scale
NIBS: Non-invasive brain stimulation
PROM: Passive range of motion
rTMS: Repetitive transcranial magnetic stimulation
SAEs: Serious adverse events
SCP: Spastic cerebral palsy
tDCS: Transcranial direct current stimulation
TENS: Transcutaneous electrical nerve stimulation
tPCS: Transcranial pulsed current stimulation
tsESS: Transcutaneous electrical stimulation of the spine
