# Supplementary material for: The effect of combined transcranial pulsed current stimulation and transcutaneous electrical nerve stimulation on lower limb spasticity in children with spastic cerebral palsy: a randomized and controlled clinical study
Source: BMC Pediatr. 2021 Mar 24;21:141. doi: 10.1186/s12887-021-02615-1 (PMC7989146; doi:10.1186/s12887-021-02615-1)
Supplement: Supplementary file 2 — Additional file 2. Trial Protocol. [file 12887_2021_2615_MOESM2_ESM.pdf]

The effect of combined transcranial pulsed current stimulation and transcutaneous electrical nerve stimulation on lower limb spasticity in children with spastic cerebral palsy classified as GMFCS Levels III–V: A randomized and controlled clinical study

Short Title:
Effect of combined tPCS and TENS on spasticity in children with spastic CP in GMFCS Levels III-V.

Ethics Committee Process No: 20181210

Chinese Clinical Trial Registry Registration No: ChiCTR1800020283
Date of Registration: 22 December 2018

Principal Investigator: Liu Zhen Huan

Primary Sponsor: Nanhai Maternity and Children's Hospital Affiliated to Guangzhou University of Traditional Chinese Medicine

Secondary Sponsor: Guangzhou Social Welfare Institute Rehabilitation Hospital

Funded by: Guangzhou Yirui Charitable Foundation

Section 1: Background of Study

Cerebral palsy is the most common cause of motor disability in childhood and has a worldwide incidence of approximately 2 to 2.5 cases per 1,000 live-born infants. Spastic cerebral palsy (SCP) is the most common CP subtype, accounting for 77% of all cases of cerebral palsy [1], typically presenting increased muscle tone, hyperreflexia, exaggerated deep tendon reflexes, and, in some cases, clonus [2]. In the sub-category of SCP children who have severe spastic diplegia and spastic quadriplegia, categorized on Level III and levels VI–V as according to the Gross Motor Function Classification System (GMFCS) [3], majority have both legs as the most affected limbs. Spasticity often results in the development of muscle and joint contractures, torsional deformities of bone, and joint instability at the hip, knee and ankle [4], which can impact wheelchair positioning, transfers, dressing, hygiene and increase the burden of caregiving. Thus, treating lower limb spasticity is an important rehabilitation goal for SCP children categorized as GMFCS Level III–V, for the purposes of improved comfort, reduced pain, slower progression of musculoskeletal deformities and easing of caretaker burden.

Spasticity in CP has been suggested to be due to the loss of descending inhibitory input to the dorsal reticulospinal and corticospinal tracts caused by damaged cortical motoneurons, leading to an exaggeration of excitatory inputs from the medial reticulospinal and vestibulospinal tracts, ultimately resulting in spasticity [5,6]. Current interventions for spasticity in children with SCP include oral medications, physical and occupational therapy, splinting and casting, botulinum toxin injections and surgical methods such as selective dorsal rhizotomy and intrathecal baclofen. However, many of the above methods are associated with undesirable side effects and even serious adverse events [7,8,9]. There is a pressing need for the development of new spasticity treatments, with priority given to conservative measures with the fewest side effects suitable for the long-term rehabilitation needs of children with SCP in GMFCS levels III–V. Non-invasive neuromodulation (NINM) such as non-invasive brain stimulation and afferent electrical stimulation have been proposed as possible mechanisms to manage spasticity, for example NIBS can be used repeatedly to target cortical stimulation, activating or inhibiting neural activity in the targeted regions of the cortex [10].

1.1 Physical therapy

Physical therapy is a recommended method to always be present in any rehabilitative program for spasticity, while other methods may be adjunct treatments used to achieve

individualized, patient-centered goals set collaboratively with patients and their caregivers [11]. However, for children with spastic cerebral palsy categorized on GMFCS Levels III-V, due to the frequent existence of comorbidities such as intellectual and cognitive dysfunctions, physiotherapy methods requiring an active participation from the child such as Bobath therapy [12] and constraint-induced therapy [13] will likely be difficult to execute, thus passive approaches such as passive muscle stretching and massage are deemed more suitable. Evidence supports massage (Chinese Tui Na) as an intervention to improve blood and lymphatic circulation, enhance inelastic and elastic properties of muscles and connective tissue and in particular directed towards reducing stretch reflex responsible for hypertonia [14].

1.2 Transcranial Pulsed Current Stimulation

Transcranial pulsed current stimulation (tPCS) is a type of noninvasive brain stimulation (NIBS) modality that has recently gained increasing attention in experimental settings [15-25], delivering pulsed currents at a predetermined frequency to the cortex, as opposed to the direct current provided by transcranial direct current stimulation (tDCS). During treatment, tPCS acts with a weak current typically with intensity < 2mA, to influence the perturbation of cortical neurons through surface gel electrodes (anodes and cathodes) placed on the scalp. In healthy subjects, Jaberzadeh et al [15] demonstrated greater effects on corticospinal excitability of the primary motor cortex with anodal tPCS of short inter-pulse-interval as compared to tDCS. Even though the physiological mechanisms underpinning the induction of corticospinal excitability by tPCS are not yet understood, it is assumed that this new neuromodulation modality induces its effects through a combination of tonic and phasic effects, i.e. facilitating an increased/decreased rate of spontaneous neuronal firing through the depolarization/ hyperpolarization of the local resting membrane potential of cells as well as through the phasic effect from on–off nature of pulses on voltage gated carrier proteins in the membranes of M1 neurons [16]. tPCS has also been demonstrated to increase the power and connectivity of endogenously generated brain oscillation in a frequency-specific manner [17,21-22,24-25] and has an intensity-dependent facilitatory effect on interhemispheric connectivity [20]. Furthermore, tPCS research based on head modeling simulation also suggested that it may influence deeper brain structures such as the brain stem, the thalamus and the hypothalamus rather than simply increasing cortical excitability of surface neurons [26]. Currently, no studies have been done involving tPCS for the treatment of SCP children but the safety of tPCS has been investigated in the treatment of Parkinson's' disease, with

much success in improving gait and balance post-treatment [27]. The possibility that tPCS could influence functional connectivity in brain networks and reach subcortical brain regions could be significant for children categorized on GMFCS Levels III – V, where many suffered damage to both sides of their brain and have highly compromised neurotransmission.

1.3 Transcutaneous Electrical Nerve Stimulation

Transcutaneous electrical nerve stimulation (TENS) is a common form of non-invasive neuromodulation (NINM) that has been used for many years, either as a stand-alone or adjunct therapy, in the rehabilitation of children with CP. TENS involves the application of electric currents onto the skin using surface electrodes to target spastic muscles and/or their antagonists [28,29]. The reduction of spasticity caused by TENS is purportedly due to the massive recruitment of sensory afferents that can suppress motoneuronal excitability through the depression of propriospinal interneurons or the induction of long-term synaptic changes in primary afferents in the dorsal horn [30]. TENS can be applied to the spine and is also known as transcutaneous electrical stimulation of the spine w(tsESS) [31-34]; Application of tsESS to the cervicothoracic and thoracolumbar regions have been observed to influence the spinal pathways leading to normalization of spinal reflex hyperexcitability and treatment of hypertonia in subjects with lesions to upper motor neurons [33]. More commonly, TENS is used directly on affected muscles in CP. In a study involving 27 ambulant children with spastic diplegic CP [35], 100hz TENS was shown to be able to significantly decrease hip adductor spasticity after both a single trial and one-week trial, though there was no significant difference in the level of improvement in spasticity between the two test periods. In a small study by Katz et al. [36], five children with diplegic and hemiplegic type CP completed three months of 20 Hz electrical stimulation to the quadricep muscle at home, 30 minutes per day, for at least five days per week, the Intervention Group showed a significant increase in average movement speed, as well as decreased knee jerk (as a measure of spasticity) and knee torque impulse, when compared to the Control Group. In a study by Aray et al. [37], the effect of 20–40 Hz TENS was tested on five ambulant children with hemiplegic and diplegic CP who underwent electrical stimulation to the quadricep femoris (QF) and anterior tibialis (TA) for 30 min a session, four to five times a week, for four weeks, in addition to standard physical therapy. Significant improvements in walking speed and cadence were demonstrated in the Intervention Group when compared to the Control Group; however, post treatment MAS and H-reflex measurements using EMG parameters on QF and TA between groups

were not significant. Thus, the efficacy of TENS in the treatment of spasticity seems to depend on its parameters. TENS that use high frequencies (≥ 99 Hz) could recruit larger diameter afferents and have been reported to relieve spasticity accompanied by a decrease on H-reflex amplitude which was not observed when lower frequencies (< 50 Hz) were used [38-40]. Other than apparent pulse frequency, power harmonics at higher frequency bandwidth of the stimulation waveform also displayed a trend of increasing correlation with the induced force enhancements that contributed to corticospinal neuromodulation in an animal model study of peripheral electrical stimulation [41], with highest correlation observed in the frequency spectrum of 400-480hz.

1.4 Combined scalp stimulation (tPCS) and peripheral stimulation (TENS)

No studies were found using a combination of scalp and peripheral stimulation for the treatment of spasticity in children with SCP. In Chinese traditional therapies, it was reported in a study by LZH et al [42], that "Tong Du Xing Shen" acupuncture methodology, involving a concurrent stimulation of acupoints on the scalp, along the governing vessel (spine), and on targeted lower limb muscles, could significantly improve spasticity, motor function and cognition in children with more severe forms of SCP categorized on GMFCS Levels III, IV and V. Acupoints are believed to possess special electrical properties with increased conductance, reduced resistance, and elevated electrical potential [43], thus acupoint positions may be a good reference for NINM stimulation sites. In other medical conditions related to chronic pain, chronic stroke, and spinal cord injury , several studies have reported enhanced benefits of combining transcranial and peripheral stimulation that seemed to surpass levels reached by single intervention alone [44-49]. Celnik et al. [44] tested the effects of combined tDCS and peripheral nerve stimulation (PNS) on the ability to perform finger motor sequences with the paretic hand in chronic stroke patients. The study found that the combined protocol resulted in a marked improvement in the number of correct key presses relative to other test groups (i.e., PNS_{sham} and $tDCS_{sham}$, tDCS and PNS_{Sham}, PNS and $tDCS_{Sham}$). In a case report of a chronic stroke patient who suffered from severe right hemiparesis, Satow et al. [44] showed that the effects of a combined stimulation using cathodal tDCS and 50 Hz peripheral electrical stimulation on the lower limbs brought about a surprising improvement in gait ability in a chronic stage of stroke which was maintained for at least one month after intervention. Boggio et al. [46] showed that the combination of TENS with tDCS is more effective, when compared with tDCS alone and sham stimulation,

in the reduction of chronic pain of the arms. Post-hoc tests showed that tDCS/TENS significantly reduced pain by 36%, while tDCS-alone reduced pain by 15.5%, and no pain reduction was observed after sham stimulation. Yamaguchi et al. [47] reported that simultaneous application of anodal tDCS and 100 Hz patterned electrical stimulation to the common peroneal nerve significantly increased changes in disynaptic reciprocal inhibition and long-latency presynaptic inhibition in individuals with spinal cord injury (SCI), and that the number of ankle movements significantly increased at 20 min after the stimulation, thus concluding that a-tDCS combined with PES could induce spinal plasticity and improve ankle movement in patients with incomplete SCI.

Both tPCS and TENS have very minimal side effects, are relatively low in cost and can be easily administered even in the presence of comorbidities such as severe intellectual disability that often exist in children with SCP categorized as GMFCS levels III–V [50]. Given supporting evidence on the induction of summative effects on increasing corticospinal excitability when NINM modalities were combined, the concurrent application of tPCS (on scalp) and TENS (on spine and lower limbs), according to "Tong Du Xing Shen" acupuncture methodology, may present a novel, safe and effective method in improving lower limb spasticity in children with SCP categorized as GMFCS levels III–V.

Section 2: Methods/Design

2.1 Objective of the Study

The objective of the proposed study is to test the effectiveness of a combination of transcranial pulsed current stimulation (tPCS) and transcutaneous electrical nerve stimulation (TENS) as an innovative strategy for improving lower limb spasticity in children with spastic cerebral palsy (SCP) categorized on levels III–V of the Gross Motor Function Classification System (GMFCS) , who face increased challenges in spasticity management and currently lacking treatment options with low/no side effects.

2.2 Hypothesis

A combination of tPCS and TENS, applied concurrently with multiple stimulating electrodes covering the scalp, spine and lower limbs, would be effective in improving lower limb

spasticity in children with SCP categorized on GMFCS levels III–V, compared to routine physical therapy alone.

2.3 Study Design

This will be a prospective, controlled, equal randomization study (1:1), to be conducted at Guangzhou City Social Welfare Institute Rehabilitation Hospital in Guangdong Province, China (Fig 1).

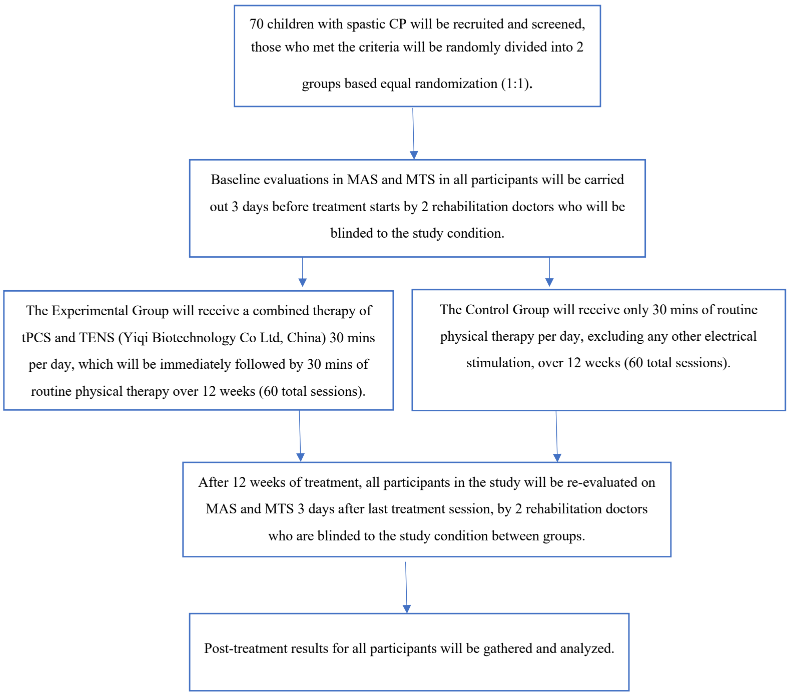

Fig 1. Flowchart of study based on Consolidated Standards of Reporting Trials.

2.4 Ethics Consideration

The present study is designed by Nanhai Maternity and Children's Hospital Affiliated to Guangzhou University of Traditional Chinese Medicine and received approval from the Clinical Research Ethics Committee of Guangzhou City Social Welfare Institute Rehabilitation Hospital under the process number 20181210. This study complies with the ethical standards established in accordance with the Declaration of Helsinki (2013 edition)

and has been registered with the Chinese Clinical Trial Registry under registration number ChiCTR1800020283. All guardians are given full right to know about all aspects of the research and those agreeing to the participation of their child will sign a written informed consent. The participants will be allowed to withdraw from the research at anytime with no negative repercussions.

2.5 Study sample and recruitment

Children with spastic CP between the ages of two and twelve years, classified on levels III, IV and V of the GMFCS, will be recruited from Guangzhou City Social Welfare Institute Rehabilitation Hospital, Guangdong, China.

2.5.1 Inclusion criteria

SCP was diagnosed according to diagnostic criteria of CP found in international guidelines [51]: children with CP aged 2–12 years of age, with GMFCS classification levels of III, IV, or V [52]; lower limb muscle tone in Grades I–IV in accordance to the Modified Ashworth Scale (MAS)[53]; no severe cardiopulmonary diseases; and voluntary participation and informed consent signed**.**

2.5.2 Exclusion criteria

Not diagnosed as spastic CP in accordance to diagnostic criteria of CP found in international guidelines [51]: children < 2 years and > 12 years of age; GMFCS classification of levels I or II [52]; lower limb muscle tone of Grade 0 in accordance with the MAS [53]; initiation of oral antispastic medication, botulinum toxin injections, or surgery performed less than 90 days before enrollment; uncontrollable epilepsy defined as the occurrence of seizures despite the use of at least one antiepileptic drug at an adequate dose; history of craniotomy or skull defects; severe neurological disorders, such as brain tumors, intracranial infection/ lesions, metal implants in the skull; Severe orthopedic deformities; and immune diseases and skin infections.

2.5.3 Drop-out criteria

Subjects who voluntarily terminated treatment during the course of treatment; subjects who did not receive treatment according to plan, either due to poor compliance or non-cooperation; and subjects who were not suitable to continue the trial due to serious adverse reactions or appearance of other accompanying diseases.

2.6 Sample size

The number of subjects needed in each study group to test the primary hypothesis was determined based on previous clinical trials assessing spasticity in cerebral palsy children. A prior study by Auvichayapat [54] testing the effects of anodal tDCS on upper limb spasticity using Modified Ashworth Scale (MAS) found that a sample size of 46 individuals divided into two groups (1 mA a-tDCS [n = 23], Sham a-tDCS [n = 23]) had an effect associated with a power of 0.90 with an alpha level of 0.05 [57]. If the combined tPCS and TENS therapy in this study had a similar effect on our primary outcome measure of MAS, the authors determined that 60 participants (30 per condition) would be sufficient to provide a power of 0.90 with an alpha of 0.05, to which we will add 10 participants to compensate for possible dropouts, totaling 70 participants.

2.7 Randomization and Allocation Concealment

Those who meet the eligibility criteria and with written consent obtained from their respective guardians to the participation of their children, will be randomly allocated to one of the two groups (Experiment Group and Control Group) using a simple random sampling method in accordance to China clinical research standards in "Methodology of Clinical Scientific Research of Integrated Traditional Chinese and Western Medicine (2nd edition)". The Experimental Group will be treated with routine physiotherapy and combined tPCS and TENS therapy. The Control Group will be treated with routine physiotherapy only.

A number sequence will be first listed in the order of 1 to n (where n represents the total number of eligible participants). Stata 11.0 software will be used to generate another set of random numbers that is each assigned to one number in this number sequence 1 to n chronologically as they are generated. The number sequence 1 to n will subsequently be sorted based on the ascending values of the assigned random number, thereby re-ordering the number sequence. The first half of the re-ordered number sequence will be allocated to the Experimental Group while the bottom half will be allocated to the Control Group. Placement cards each written with a number from 1 to n and its corresponding group (either experimental or control) will be placed into opaque envelopes and sealed to ensure the concealment of the allocation. All n envelopes will be handed over to an administrator uninvolved in the random sampling process, who will give out the envelopes to the eligible patients entering the trial following pre-intervention evaluation. Each patient will then be allocated according to the group stipulated on the placement card.

2.8 Assessment

Evaluations using Modified Ashworth Scale (MAS) and Modified Tardieu Scale (MTS) will be conducted on all participants by two qualified rehabilitation doctors who have been involved in clinical work of pediatric rehabilitation for more than five years. The evaluators are blinded to the allocation of the children to the two groups and did not participate in the treatment of the subjects. A baseline evaluation and a post-treatment evaluation will be held. Both baseline and post-treatment evaluation will be conducted in a designated evaluation room that is spacious, quiet and bright, as follows:

- Baseline evaluation: three days prior to intervention
- Post-treatment evaluation: three days following last session of treatment
- Each scale will be repeatedly measured 3 times each time. Each score was the average of the three measurements.

2.8.1 Modified Ashworth Scale (MAS) [53]

The MAS was used to evaluate muscle spasticity in the lower limbs. MAS evaluation involves the rater manually moving a limb through the range of motion to passively stretch specific muscle groups and a six-point ordinal scale for grading the resistance encountered during such passive muscle stretching. MAS grades of spasticity are as follows: 0 = normal muscle tone; 1 = slight increase in muscle tone, manifested by catch and release or by minimal resistance at the end; 1+ = slight increase in muscle tone, manifested by a catch, followed by minimal resistance throughout; 2 = more marked increase in muscle tone, but limb easily flexed; 3 = considerable increase in muscle tone, passive movement difficult; and 4 = limb rigid in flexion or extension.

Modified Ashworth Scale

Grade	Description
0	No increase in muscle tone
1	Slight increase in muscle tone, manifested by a catch and release or by minimal resistance at the end of the range of motion when the affected part(s) is moved in flexion or extension
1+	Slight increase in muscle tone, manifested by a catch, followed by minimal resistance throughout the remainder (less than half) of the Range of Movement
2	More marked increase in muscle tone through most of the Range of Movement, but affected part(s) easily moved
3	Considerable increase in muscle tone, passive movement difficult

4	Affected part(s) rigid in flexion or extension

2.8.2 Modified Tardieu Scale (MTS) [55,56]

MTS will be used to evaluate the degree of spasticity in the lower limbs of participants. The MTS uses standardized procedures to measure quality of muscle reaction at specified velocities (i.e., fast stretch and slow controlled motion). During the fast stretch, the particular angle at which "catch" [57] occurs from hyperactive stretch reflex is called R1, also known as angle of muscle reaction. During the slow controlled motion, the passive range of motion (PROM) is assessed (called R2), representing the muscle length at rest and recorded as an angle. The difference between the two measures (i.e., R2 − R1; dynamic component of spasticity) is recorded as R. A large difference between R1 and R2 suggests a large dynamic component with a greater capacity for change or improvement. A small difference between R1 and R2 suggests a predominantly fixed contracture in the muscle with a poorer capacity for change.

.

Modified Tardieu Scale

Grade	Description
0	No resistance throughout the course of the passive movement.
1	Slight resistance throughout the course of the passive movement, with no clear catch at a precise angle.
2	Clear catch at a precise angle, interrupting the passive movement, followed by a release.
3	Fatigable clonus (<10 seconds when maintaining pressure) occurring at a precise angle.
4	Infatigable clonus (>>10 seconds when maintaining pressure) occurring at a precise angle.
5	Joint is immoveable

Section 3: Intervention

3.1 Combined tPCS and TENS therapy

Two therapists with more than 5 years' experience in electrical stimulation will be in charge of the treatment of subjects with the combined tPCS and TENS therapy in the Experiment group. The multichannel pulsed current stimulator (YQ-D507; Yiqi

Biotechnology Co. Ltd, China) will be used and the stimulation procedure will be carried out using six pairs of (6*9 cm) surface gel electrodes. tPCS: The first pair of electrodes will be used for cerebello-cerebral stimulation, with the anode electrode positioned over Cz (according to the 10–20 International Electroencephalogram System [60]), covering the Baihui acupoint, and cathode electrode positioned horizontally to cover the cerebellum region, coinciding with Oz (according to the 10–20 International Electroencephalogram System [58]; the bottom edge centered over the inion (Figure 2, 1+ and 1−). The skin of the scalp was required to be cleaned with saline prior to electrode placement. Stimulating current intensity was set to 1 mA. TENS: The second to sixth pairs of electrodes were used for afferent stimulation of the spinal cord and targeted muscle in the lower limbs. The second pair of electrodes was placed on the cervicothoracic region of the spine, with the anode covering C6–C7 and cathode covering T1–T2 [59] (Figure 2, 2+ and 2−). The third pair of electrodes was placed on the thoracolumbar region, with the anode covering T11–12 and cathode covering L4–L5 [31, 32] (Figure 2, 3+ and 3−). The fourth pair of electrodes were placed on the adductor longus muscles of the lower limbs (Figure 2, 4+ and 4−). The fifth pair of electrode pads (Figure 2, 5+ and 5-) were placed on the rectus femoris muscles of the lower limbs. The sixth pair of electrode pads (Figure 2, 6+ and 6-) were placed on the gastrocnemius muscles of the lower limbs. The specific strength of the second to sixth pairs of electrodes was adjusted according to the degree of tolerance of individual children, with current intensity varying from 0 to 10 mA. Time of stimulation for all six pairs is 30 minutes each session, the device has one button controlling each pair of electrodes that allows the operator to control the intensity of the current. Stimulation will be raised from 0 to the desired level and diminished to 0 in the final one second. Combined tPCS and TENS therapy will be done 5 times a week, for 12 consecutive weeks, totaling 60 sessions.

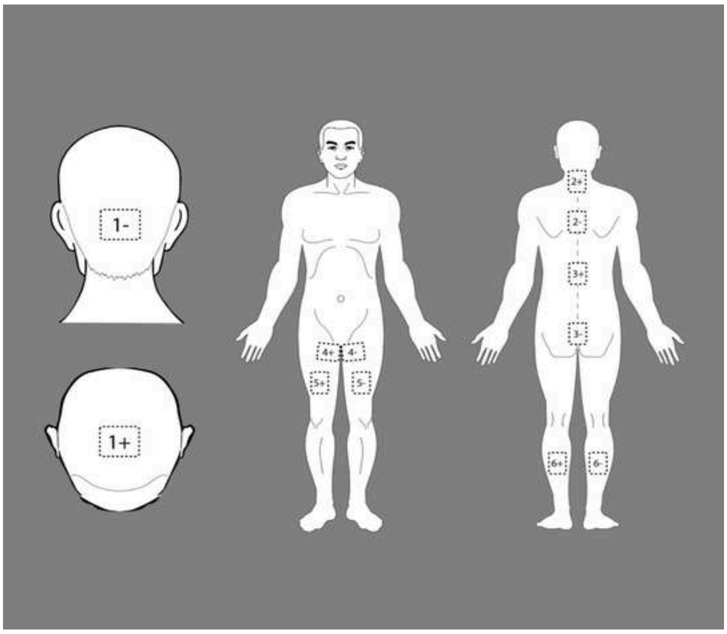

Fig 2: Position of electrodes patches during mTPCS stimulation

The output parameters of mTPCS in this study were as follows:

- tPCS (1st electrode pair) intensity: 1 mA
- TENS (2nd –6th electrode pairs) intensity: 0–10 mA
- Pulse Width of current: 140ms
- High Frequency: 400 Hz
- Waveform: Unidirectional monophasic pulse square wave

3.2 Physical therapy

Physical therapy will be done by two physical therapists with more than five years' experience and will consist of passive stretching exercises and Chinese "Tui Na" massages [14] often used for children with SCP to reduce muscle stiffness, with a focus on lower limb muscles, it will not include other forms of electrical stimulation. Each physical therapy session will be 30 minutes, carried out five times a week over twelve consecutive weeks. Physical therapy for the Experiment Group will be administered immediately after combined tPCS and TENS therapy, while the Control group will be given only physical therapy.

Section 4: Safety Considerations

Safety issues will be identified a priori by the authors who, on average, have over 20 years of pediatric clinical experience in China. The tPCS used for cortical stimulation in the

study belonged to the category of low-intensity transcranial electrical stimulation (tES) and no serious adverse events (SAEs) have been reported so far in over 18,000 sessions of low-intensity tES administered to healthy subjects, as well as in neurological and psychiatric patients [60]. Moderate adverse events (AEs) in tES, as defined by the necessity to intervene, are rare, and include skin burns due to suboptimal electrode-skin contact. Mild side effects in tES include itching, tingling, burning sensations, and transient redness may occur during treatment [61-65]. In a systematic review by Biksom et al [66], it was reported that at least 2800 sessions of tDCS have been applied across nearly 500 child subjects covering medical conditions such as cerebral palsy, stroke, encephalitis, epilepsy, schizophrenia and attention deficit hyperactivity [67-74], and no serious adverse effects have been reported. Furthermore, a study by Jaberzadeh that compared tPCS to tDCS showed that participants tolerated a-tPCS better than the conventional a-tDCS [15].

For safety of tES on children, the recommended dose needs to compensate for thinner skull and lower resistance [75, 76]. Mattai et al. [77] explored the safety and tolerability of tDCS in children with childhood-onset schizophrenia and found that 10 sessions of 2 mA tDCS for 20 min, 25 cm electrodes, was administered without incident in the test subjects with no serious side effects. In the present study, tPCS (Unidirectional monophasic pulse square wave) will be controlled at 1mA, 30mins per session, which is within the confines of conventional tES and safe for children. In addition, our study design passed the safety review conducted by Guangzhou City Social Welfare Institute Rehabilitation Hospital Ethics committee, who gave approval for this study.

As a safety monitoring plan, two experienced pediatric nurses will be assigned to systematically observed all participants during the study for adverse reactions such as seizure, nausea, behavioral changes, or severe discomfort, and at the end of each treatment session, the children and/ or their caregivers in the Experiment group will be asked about side effects. In the event any Adverse Event occurs, there will be immediate termination of treatment and AE will be recorded and reported accordingly.

Section 5: Statistical analysis

SPSS v. 20.0 will be used for statistical analysis of the data. The data will be entered into Excel tables by double entry and checking to establish the database. The data of MAS and

MTS scales will be described in terms of means ± standard deviation. If the data of MAS , and MTS scale did not conform to the conditions of normal distribution and homogeneity of variance, the Mann-Whitney test will be used; if the data of MAS and MTS scale did conformed to the conditions of normal distribution and homogeneity of variance, then independent sample t-test will be used. Pearson correlation analysis will be used for the correlation analysis between the GMFCS grade, gender and age and muscle tone after treatment in the Experiment Group. Either one-way ANOVA or Chi-squared test will be used for inter-group data comparison. A p-value less than 0.05 will be considered statistically significant.

References

[1] Yeargin-Allsopp M, Van Naarden Braun K, Doernberg NS, Benedict RE, Kirby RS, Durkin MS. Prevalence of cerebral palsy in 8-year-old children in three areas of the United States in 2002: a multisite collaboration. *Pediatrics*. 2008;121(3):547-554. doi:10.1542/peds.2007-1270

[2] Baird HW, Gordon EC. (1983) *Neurological Evaluation of Infants and Children.* Suffolk: Lavenham Press.

[3] Palisano R, Rosenbaum P, Walter S, Russell D, Wood E, Galuppi B. Development and reliability of a system to classify gross motor function in children with cerebral palsy. *Dev Med Child Neurol*. 1997;39(4):214-223. doi:10.1111/j.1469-8749.1997.tb07414

[4] Gormley ME Jr, Krach LE, Piccini L. *Spasticity management of the child with spastic quadriplegia.* Eur J Neurol *5(Suppl 5):S127-S135.*

[5] Brown P. Pathophysiology of spasticity. *J Neurol Neurosurg Psychiatry*. 1994;57(7):773-777. doi:10.1136/jnnp.57.7.773

[6] Goldstein EM. Spasticity management: an overview. J Child Neurol. 2001;16(1):16-23. doi:10.1177/088307380101600104

[7] Montané E, Vallano A, Laporte JR. Oral antispastic drugs in nonprogressive neurologic diseases: a systematic review. *Neurology*. 2004;63(8):1357-1363. doi:10.1212/01.wnl.0000141863.52691.44

[8] Hoving MA, van Raak EP, Spincemaille GH, et al. Safety and one-year efficacy of intrathecal baclofen therapy in children with intractable spastic cerebral palsy. *Eur J Paediatr Neurol*. 2009;13(3):247-256. doi:10.1016/j.ejpn.2008.05.002

[9] Borrini L, Bensmail D, Thiebaut JB, Hugeron C, Rech C, Jourdan C. Occurrence of adverse events in long-term intrathecal baclofen infusion: a 1-year follow-up study of 158 adults. *Arch Phys Med Rehabil*. 2014;95(6):1032-1038. doi:10.1016/j.apmr.2013.12.019

[10] Chung MG, Lo WD. Noninvasive brain stimulation: the potential for use in the rehabilitation of pediatric acquired brain injury. *Arch Phys Med Rehabil*. 2015;96(4 Suppl):S129-S137. doi:10.1016/j.apmr.2014.10.013

[11] Stiens SA, O'Young BJ, Young MA. Person-centered rehabilitation. In: O'Young BJ, Young MA, Stiens SA, editors. Physical Medicine & Rehabilitation Secrets. 3rd ed. Philadelphia:Mosby Elsevier; 2008. pp. 118–25.

[12] Brown GT, Burns SA: The efficacy of neurodevelopmental treatments in children: a systematic review. *Br J Occup Ther* 2001, 64:235-244.

[13] Zafer H, Amjad I, Malik AN, Shaukat E. Effectiveness of Constraint induced movement therapy as compared to bimanual therapy in Upper motor function outcome in child with hemiplegic Cerebral palsy. *Pak J Med Sci*. 2016;32(1):181–184. doi:10.12669/pjms.321.8491

[14] Zhou X-j, Zheng K. Treatment of 140 cerebral palsied children with a combined method based on traditional Chinese medicine (TCM) and western medicine. J Zhejiang Univ Sci B. 2005; 6:57-60.

[15] Jaberzadeh S, Bastani A, Zoghi M. Anodal transcranial pulsed current stimulation: A novel technique to enhance corticospinal excitability. *Clin Neurophysiol*. 2014;125(2):344-351. doi:10.1016/j.clinph.2013.08.025

[16] Jaberzadeh S, Bastani A, Zoghi M, Morgan P, Fitzgerald PB. Anodal Transcranial Pulsed Current Stimulation: The Effects of Pulse Duration on Corticospinal Excitability. *PLoS One*. 2015;10(7):e0131779. Published 2015 Jul 15. doi:10.1371/journal.pone.0131779

[17] Castillo Saavedra L, Morales-Quezada L, Doruk D, et al. QEEG indexed frontal connectivity effects of transcranial pulsed current stimulation (tPCS): A sham-controlled mechanistic trial. *Neurosci Lett*. 2014;577:61-65. doi:10.1016/j.neulet.2014.06.021

[18] Saito K, Otsuru N, Inukai Y, et al. Comparison of transcranial electrical stimulation regimens for effects on inhibitory circuit activity in primary somatosensory cortex and tactile spatial discrimination performance. *Behav Brain Res*. 2019;375:112168. doi:10.1016/j.bbr.2019.112168

[19] Vasquez A, Malavera A, Doruk D, et al. Duration Dependent Effects of Transcranial Pulsed Current Stimulation (tPCS) Indexed by Electroencephalography. *Neuromodulation*. 2016;19(7):679-688. doi:10.1111/ner.12457

[20] Vasquez AC, Thibaut A, Morales-Quezada L, Leite J, Fregni F. Patterns of brain oscillations across different electrode montages in transcranial pulsed current stimulation. *Neuroreport*. 2017;28(8):421-425. doi:10.1097/WNR.0000000000000772

[21] Thibaut A, Russo C, Hurtado-Puerto AM, et al. Effects of Transcranial Direct Current Stimulation, Transcranial Pulsed Current Stimulation, and Their Combination on Brain Oscillations in Patients with Chronic Visceral Pain: A Pilot Crossover Randomized Controlled Study. *Front Neurol*. 2017;8:576. Published 2017 Nov 1. doi:10.3389/fneur.2017.00576

[22] Thibaut A, Russo C, Morales-Quezada L, et al. Neural signature of tDCS, tPCS and their combination: Comparing the effects on neural plasticity. *Neurosci Lett*. 2017;637:207-214. doi:10.1016/j.neulet.2016.10.026

[23] Ma Z, Du X, Wang F, et al. Cortical Plasticity Induced by Anodal Transcranial Pulsed Current Stimulation Investigated by Combining Two-Photon Imaging and Electrophysiological Recording. *Front Cell Neurosci*. 2019;13:400. Published 2019 Aug 29. doi:10.3389/fncel.2019.00400

[24] Morales-Quezada L, Leite J, Carvalho S, Castillo-Saavedra L, Cosmo C, Fregni F. Behavioral effects of transcranial pulsed current stimulation (tPCS): Speed-accuracy tradeoff in attention switching task. *Neurosci Res*. 2016;109:48-53. doi:10.1016/j.neures.2016.01.009

[25] Singh A, Trapp NT, De Corte B, et al. Cerebellar Theta Frequency Transcranial Pulsed Stimulation Increases Frontal Theta Oscillations in Patients with Schizophrenia. *Cerebellum*. 2019;18(3):489-499. doi:10.1007/s12311-019-01013-9

[26] Datta A, Dmochowski JP, Guleyupoglu B, Bikson M, Fregni F. Cranial electrotherapy stimulation and transcranial pulsed current stimulation: a computer based high-resolution modeling study. *Neuroimage*. 2013;65:280-287. doi:10.1016/j.neuroimage.2012.09.062

[27] Alon G, Yungher DA, Shulman LM, Rogers MW. Safety and immediate effect of noninvasive transcranial pulsed current stimulation on gait and balance in Parkinson disease. *Neurorehabil Neural Repair*. 2012;26(9):1089-1095. doi:10.1177/1545968312448233

[28] Goulet C, Arsenault AB, Bourbonnais D, Laramée MT, Lepage Y. Effects of transcutaneous electrical nerve stimulation on H-reflex and spinal spasticity. *Scand J Rehabil Med*. 1996;28(3):169-176.

[29] Potisk KP, Gregoric M, Vodovnik L. Effects of transcutaneous electrical nerve stimulation (TENS) on spasticity in patients with hemiplegia. *Scand J Rehabil Med*. 1995;27(3):169-174.

[30] Dewald JP, Given JD, Rymer WZ. Long-lasting reductions of spasticity induced by skin electrical stimulation. *IEEE Trans Rehabil Eng*. 1996;4(4):231-242. doi:10.1109/86.547923

[31] Minassian K, Persy I, Rattay F, Dimitrijevic MR, Hofer C, Kern H. Posterior root-muscle reflexes elicited by transcutaneous stimulation of the human lumbosacral cord. *Muscle Nerve*. 2007;35(3):327-336. doi:10.1002/mus.20700

[32] Sabbahi MA, Sengul YS. Thoracolumbar multisegmental motor responses in the upper and lower limbs in healthy subjects. *Spinal Cord*. 2011;49(6):741-748. doi:10.1038/sc.2010.165

[33] Knikou M. Transpinal and transcortical stimulation alter corticospinal excitability and increase spinal output. *PLoS One*. 2014;9(7):e102313. Published 2014 Jul 9. doi:10.1371/journal.pone.0102313

[34] Nardone R, Höller Y, Taylor A, et al. Noninvasive Spinal Cord Stimulation: Technical Aspects and Therapeutic Applications. *Neuromodulation*. 2015;18(7):580-591. doi:10.1111/ner.12332

[35] Alabdulwahab SS, Al-Gabbani M. Transcutaneous electrical nerve stimulation of hip adductors improves gait parameters of children with spastic diplegic cerebral palsy. *NeuroRehabilitation*. 2010;26(2):115-122. doi:10.3233/NRE-2010-0542

[36] Katz A, Tirosh E, Marmur R, Mizrahi J. Enhancement of muscle activity by electrical stimulation in cerebral palsy: a case-control study. *J Child Neurol*. 2008;23(3):259-267. doi:10.1177/0883073807308695

[37] Arya BK, Mohapatra J, Subramanya K, Prasad H, Kumar R, Mahadevappa M. Surface EMG analysis and changes in gait following electrical stimulation of quadriceps femoris and tibialis anterior in children with spastic cerebral palsy. *Conf Proc IEEE Eng Med Biol Soc*. 2012;2012:5726-5729. doi:10.1109/EMBC.2012.6347295

[38] Garcia MAC, Vargas CD. Is somatosensory electrical stimulation effective in relieving spasticity? A systematic review. *J Musculoskelet Neuronal Interact*. 2019;19(3):317-325.

[39] Levin MF, Hui-Chan CW. Relief of hemiparetic spasticity by TENS is associated with improvement in reflex and voluntary motor functions. *Electroencephalogr Clin Neurophysiol*. 1992;85(2):131-142. doi:10.1016/0168-5597(92)90079-q

[40] Aydin G, Tomruk S, Keleş I, Demir SO, Orkun S. Transcutaneous electrical nerve stimulation versus baclofen in spasticity: clinical and electrophysiologic comparison. *Am J Phys Med Rehabil*. 2005;84(8):584-592. doi:10.1097/01.phm.0000171173.86312.69
[41] Chen CF, Bikson M, Chou LW, et al. Higher-order power harmonics of pulsed electrical stimulation modulates corticospinal contribution of peripheral nerve stimulation. *Sci Rep*. 2017;7:43619. Published 2017 Mar 3. doi:10.1038/srep43619

[42] 刘振寰,祁岩超,潘佩光等.通督醒神针刺法对脑瘫患儿头颅 CT 及 SPECT 的临床观察[C].//第七届上海国际针灸临床与科研学术研讨会论文集.2008:176-178.

[43] Ahn AC, Martinsen OG. Electrical characterization of acupuncture points: technical issues and challenges. *J Altern Complement Med*. 2007;13(8):817-824. doi:10.1089/acm.2007.7193

[44] Celnik P, Paik NJ, Vandermeeren Y, Dimyan M, Cohen LG. Effects of combined peripheral nerve stimulation and brain polarization on performance of a motor sequence task after chronic stroke. *Stroke*. 2009;40(5):1764-1771. doi:10.1161/STROKEAHA.108.540500

[45] Satow T, Kawase T, Kitamura A, et al. Combination of Transcranial Direct Current Stimulation and Neuromuscular Electrical Stimulation Improves Gait Ability in a Patient in Chronic Stage of Stroke. *Case Rep Neurol*. 2016;8(1):39-46. Published 2016 Feb 12. doi:10.1159/000444167

[46] Boggio PS, Amancio EJ, Correa CF, et al. Transcranial DC stimulation coupled with TENS for the treatment of chronic pain: a preliminary study. *Clin J Pain*. 2009;25(8):691-695. doi:10.1097/AJP.0b013e3181af1414

[47] Yamaguchi T, Fujiwara T, Tsai YA, et al. The effects of anodal transcranial direct current stimulation and patterned electrical stimulation on spinal inhibitory interneurons and motor function in patients with spinal cord injury. *Exp Brain Res*. 2016;234(6):1469-1478. doi:10.1007/s00221-016-4561-4

[48] Houde F, Harvey MP, Tremblay Labrecque PF, Lamarche F, Lefebvre A, Leonard G. Combining Transcranial Direct Current Stimulation and Transcutaneous Electrical Nerve Stimulation to Relieve Persistent Pain in a Patient Suffering from Complex Regional Pain Syndrome: A Case Report. *J Pain Res*. 2020;13:467-473. Published 2020 Mar 2. doi:10.2147/JPR.S226616

[49] Hazime FA, Baptista AF, de Freitas DG, et al. Treating low back pain with combined cerebral and peripheral electrical stimulation: A randomized, double-blind, factorial clinical trial. *Eur J Pain*. 2017;21(7):1132-1143. doi:10.1002/ejp.1037

[50] Parette HP Jr, Holder LF, Sears JD. Correlates of therapeutic progress by infants with cerebral palsy and motor delay. *Percept Mot Skills*. 1984;58(1):159-163. doi:10.2466/pms.1984.58.1.159

[51] Bax M, Goldstein M, Rosenbaum P, et al. Proposed definition and classification of cerebral palsy, April 2005. *Dev Med Child Neurol*. 2005;47(8):571-576. doi:10.1017/s001216220500112x

[52] Palisano R, Rosenbaum P, Walter S, Russell D, Wood E, Galuppi B. Development and reliability of a system to classify gross motor function in children with cerebral palsy. *Dev Med Child Neurol*. 1997;39(4):214-223. doi:10.1111/j.1469-8749.1997.tb07414.x

[53] Bohannon RW, Smith MB. Interrater reliability of a modified Ashworth scale of muscle spasticity. *Phys Ther*. 1987;67(2):206-207. doi:10.1093/ptj/67.2.206

[54] Auvichayapat P, Areeuea B, Auvichayapat N, Phuttharak W, Janyacharoen T, Tunkamnerdthai O, et al. Transient changes in brain metabolites after transcranial direct current stimulation in spastic cerebral palsy: A pilot study. Front Neurol. 2017;8:366.doi:10.3389/fneur.2017.00366

[55] Boyd, R.N. and Graham, H.K. (1999), Objective measurement of clinical findings in the use of botulinum toxin type A for the management of children with cerebral palsy. European Journal of Neurology, 6: s23-s35. doi:10.1111/j.1468-1331.1999.tb00031.x

[56] 董尚胜, 陈艳娟. 改良 Tardieu 量表在脑性瘫痪儿童下肢的应用研究[J]. 实用医学杂志, 2016, 32(16):2711-2713.

[57] Levin MF, Feldman AG. The role of stretch reflex threshold regulation in normal and impaired motor control. *Brain Res*. 1994;657(1-2):23-30. doi:10.1016/0006-8993(94)90949-0

[58] Klem GH, Lüders HO, Jasper HH, Elger C. The ten-twenty electrode system of the International Federation. The International Federation of Clinical Neurophysiology. *Electroencephalogr Clin Neurophysiol Suppl*. 1999;52:3-6.

[59] Einhorn J, Li A, Hazan R, Knikou M. Cervicothoracic multisegmental transpinal evoked potentials in humans. *PLoS One*. 2013;8(10):e76940. Published 2013 Oct 7. doi:10.1371/journal.pone.0076940

[60] Antal A, Alekseichuk I, Bikson M, et al. Low intensity transcranial electric stimulation: Safety, ethical, legal regulatory and application guidelines. *Clin Neurophysiol*. 2017;128(9):1774-1809. doi:10.1016/j.clinph.2017.06.001

[61] Brunoni, A. R., Amadera, J., Berbel, B., Volz, M. S., Rizzerio, B. G., and Fregni, F. (2011b). A systematic review on reporting and assessment of adverse effects associated with transcranial direct current stimulation. Int. J. Neuropsychopharmacol. 14, 1133–1145. doi: 10.1017/S1461145710001690

[62] Iyer, M., Mattu, U., Grafman, J., Lomarev, M., Sato, S., and Wassermann, E. (2005). Safety and cognitive effect of frontal DC brain polarization in healthy individuals. Neurology 64, 872–875. doi: 10.1212/01.WNL.0000152986.07469.E9

[63] Poreisz, C., Boros, K., Antal, A., and Paulus, W. (2007). Safety aspects of transcranial direct current stimulation concerning healthy subjects and patients. Brain Res. Bull. 72, 208–214. doi: 10.1016/j.brainresbull.2007.01.004

[64] Plazier, M., Joos, K., Vanneste, S., Ost, J., and De Ridder, D. (2012). Bifrontal and bioccipital transcranial direct current stimulation (tDCS) does not induce mood changes in healthy volunteers: a placebo controlled study. Brain Stimul. 5, 454–461. doi: 10.1016/j.brs.2011.07.005

[65] Fregni, F., Gimenes, R., Valle, A. C., Ferreira, M. J., Rocha, R. R., Natalle, L., et al. (2006c). A randomized, sham-controlled, proof of principle study of transcranial direct current stimulation for the treatment of pain in fibromyalgia. Arthr. Rheum. 54, 3988–3998. doi: 10.1002/art.22195

[66] Bikson M, Grossman P, Thomas C. Safety of transcranial Direct Current Stimulation: Evidence Based Update 2016. Brain Stimul. 2016 Sep-Oct;9(5):641-661. doi: 10.1016/j.brs.2016.06.004.

[67] Cosmo C, Baptista AF, de Sena EP. Contribution of transcranial direct current stimulation on inhibitory control to assess the neurobiological aspects of attention deficit hyperactivity disorder: randomized controlled trial. JMIR Res Protoc. 2015; 4:e56.

[68] Auvichayapat N, Rotenberg A, Gersner R, Ngodklang S, Tiamkao S, Tassaneeyakul W, et al.Transcranial direct current stimulation for treatment of refractory childhood focal epilepsy. Brain Stimulat. 2013; 6:696–700.

[69] Gillick BT, Feyma T, Menk J, Usset M, Vaith A, Wood TJ, et al. Safety and feasibility of transcranial direct current stimulation in pediatric hemiparesis: randomized controlled preliminary study. Phys Ther. 2015; 95:337–349.

[70]Young SJ, Bertucco M, Sheehan-Stross R, Sanger TD. Cathodal transcranial direct current stimulation in children with dystonia: a pilot open-label trial. J Child Neurol. 2013; 28:1238–1244.

[71] Duarte, N de AC.; Grecco, LAC.; Galli, M.; Fregni, F.; Oliveira, CS. Effect of transcranial direct current stimulation combined with treadmill training on balance and functional performance in children with cerebral palsy: a double-blind randomized controlled trial. PloS One. 2014;9:e105777.

[72] Grecco LAC, E Mendonça M, Duarte NAC, Zanon N, Fregni F, Oliveira CS. Transcranial Direct Current Stimulation Combined with Treadmill Gait Training in Delayed Neuro-psychomotor Development. J Phys Ther Sci. 2014; 26:945–950.

[73] Aree-uea B, Auvichayapat N, Janyacharoen T, Siritaratiwat W, Amatachaya A, Prasertnoo J, et al. Reduction of spasticity in cerebral palsy by anodal transcranial direct current stimulation. J Med Assoc Thail Chotmaihet Thangphaet. 2014; 97:954–962.

[74] San-Juan D, Calcáneo J de DDC, González-Aragón MF, Bermúdez Maldonado L, Avellán AM,Argumosa EVG, et al. Transcranial direct current stimulation in adolescent and adult Rasmussen's encephalitis. Epilepsy Behav EB. 2011; 20:126–131.

[75] Kessler, S. K., Minhas, P., Woods, A. J., Rosen, A., Gorman, C., and Bikson, M. (2013). Dosage considerations for transcranial direct current stimulation in children: a computational modeling study. PLoS ONE 8:e76112. doi: 10.1371/journal.pone.0076112

[76] Gillick BT, Kirton A, Carmel JB, Minhas P, Bikson M. Pediatric stroke and transcranial direct current stimulation: methods for rational individualized dose optimization. Front Hum Neurosci.2014; 8:739.

[77] Mattai A, Miller R, Weisinger B, Greenstein D, Bakalar J, Tossell J, et al. Tolerability of transcranial direct current stimulation in childhood-onset schizophrenia. Brain Stimulat. 2011;4:275–280.
